# Repurposing nitric oxide donating drugs in cancer therapy through immune modulation

**DOI:** 10.1186/s13046-022-02590-0

**Published:** 2023-01-14

**Authors:** Chung-Yen Li, Gangga Anuraga, Chih-Peng Chang, Tzu-Yang Weng, Hui-Ping Hsu, Hoang Dang Khoa Ta, Pei-Fang Su, Pin-Hsuan Chiu, Shiang-Jie Yang, Feng-Wei Chen, Pei-Hsuan Ye, Chih-Yang Wang, Ming-Derg Lai

**Affiliations:** 1 College of Medicine, Institute of basic medical sciences, National Cheng Kung University, Tainan, Taiwan, ROC; 2 PhD Program for Cancer Molecular Biology and Drug Discovery, College of Medical Science and Technology, Taipei Medical University and Academia Sinica, Taipei, Taiwan, ROC; 3 Department of Statistics, Faculty of Science and Technology, Universitas PGRI Adi Buana, Surabaya, Indonesia; 4 Department of Microbiology and Immunology, College of Medicine, National Cheng Kung University, Tainan, Taiwan, ROC; 5 Department of Surgery, National Cheng Kung University Hospital, College of Medicine, National Cheng Kung University, Tainan, Taiwan, ROC; 6 Department of Statistics, National Cheng Kung University, Tainan, Taiwan, ROC; 7 The Center for Quantitative Sciences, Clinical Medicine Research Center, National Cheng Kung University Hospital, College of Medicine, National Cheng Kung University, Tainan, Taiwan, ROC; 8 Department of Biochemistry and Molecular Biology, College of Medicine, National Cheng Kung University, Tainan, Taiwan, ROC; 9 TMU Research Center of Cancer Translational Medicine, Taipei Medical University, Taipei, Taiwan; 10 Graduate Institute of Cancer Biology and Drug Discovery, College of Medical Science and Technology, Taipei Medical University, Taipei, Taiwan

**Keywords:** Cancer, Immune, Nitric oxide donor, SNAP, Single-cell RNA-seq, CD8^+^ cytotoxic T cells, Macrophages

## Abstract

**Background:**

Nitric oxide-releasing drugs are used for cardiovascular diseases; however, their effects on the tumor immune microenvironment are less clear. Therefore, this study explored the impact of nitric oxide donors on tumor progression in immune-competent mice.

**Methods:**

The effects of three different nitric oxide-releasing compounds (SNAP, SNP, and ISMN) on tumor growth were studied in tumor-bearing mouse models. Three mouse tumor models were used: B16F1 melanoma and LL2 lung carcinoma in C57BL/6 mice, CT26 colon cancer in BALB/c mice, and LL2 lung carcinoma in NOD/SCID mice. After nitric oxide treatment, splenic cytokines and lymphocytes were analyzed by cytokine array and flow cytometry, and tumor-infiltrating lymphocytes in the TME were analyzed using flow cytometry and single-cell RNA sequencing.

**Results:**

Low doses of three exogenous nitric oxide donors inhibited tumor growth in two immunocompetent mouse models but not in NOD/SCID immunodeficient mice. Low-dose nitric oxide donors increase the levels of splenic cytokines IFN-γ and TNF-α but decrease the levels of cytokines IL-6 and IL-10, suggesting an alteration in Th2 cells. Nitric oxide donors increased the number of CD8^+^ T cells with activation gene signatures, as indicated by single-cell RNA sequencing. Flow cytometry analysis confirmed an increase in infiltrating CD8^+^ T cells and dendritic cells. The antitumor effect of nitric oxide donors was abolished by depletion of CD8^+^ T cells, indicating the requirement for CD8^+^ T cells. Tumor inhibition correlated with a decrease in a subtype of protumor macrophages and an increase in a subset of Arg1-positive macrophages expressing antitumor gene signatures. The increase in this subset of macrophages was confirmed by flow cytometry analysis. Finally, the combination of low-dose nitric oxide donor and cisplatin induced an additive cancer therapeutic effect in two immunocompetent animal models. The enhanced therapeutic effect was accompanied by an increase in the cells expressing the gene signature of NK cell.

**Conclusions:**

Low concentrations of exogenous nitric oxide donors inhibit tumor growth in vivo by regulating T cells and macrophages. CD8^+^ T cells are essential for antitumor effects. In addition, low-dose nitric oxide donors may be combined with chemotherapeutic drugs in cancer therapy in the future.

**Supplementary Information:**

The online version contains supplementary material available at 10.1186/s13046-022-02590-0.

## Background

Cancer is controlled by unrestricted growth of tumor cells and the influence of their microenvironments, including angiogenesis and evading immune destruction [1]. Tumor-associated macrophages (TAMs) are the most abundant immune cells in the tumor microenvironment (TME), and abundant TAMs in the TME are associated with poor clinical outcomes [2, 3]. TAMs are generally classified into two groups, namely M1 macrophages and M2 macrophages. During initial carcinogenesis, M1-like polarized macrophages play a role in the elimination of more immunogenic cancer cells. As the tumor progresses, M2-like polarization of macrophages plays a protumorigenic role in the tumor microenvironment by stimulating angiogenesis, lymphangiogenesis, and cancer cell proliferation [4]. Although the M1-like/M2-like paradigm is useful, it may be too simplified. Transcriptomic analysis suggested that a broad spectrum of differentiated TAMs may exist in the tumor microenvironment.

The second most abundant immune cells in the tumor microenvironment are T cells, which act as orchestrators and effectors of immunity. CD8^+^ T cells are the most prominent antitumor cells in cancer, and CD4^+^ T cells play an important role in the maintenance and activation of the CD8^+^ T cell response [5]. CD4^+^ type 1 helper T (Th1) cells mediate the antitumor response by secreting several cytokines to activate CD8^+^ T cells, macrophages, and NK cells [4]. Interestingly, T-cell dysfunction in the tumor microenvironment shares many features with T-cell exhaustion observed in chronic viral infection [6]. Dysfunction of T cells is usually associated with high expression of inhibitory receptors and loss of the production of cytokines IFNγ, IL-2 and TNFα [7].

Nitric oxide (NO) is a common molecule that participates in many cellular functions, such as neurotransmission [8–10] and vasoconstriction [11]. Some nitric oxide donors, such as sodium nitroprusside (SNP) and isosorbide mononitrate (ISMN), have been used to control hypertension in the clinic [12]. In addition to easing hypertension, nitric oxide functions as a cancer regulator, and the role of NO in cancer is dose dependent. Abnormally high supra-physiological NO concentrations (80 μM) increase reactive nitrogen oxide species (RNS) formation, ultimately leading to DNA damage and carcinogenesis [13]. In vitro experiments have suggested that nitric oxide has an inhibitory function on immune cells and endothelial cell activation [14]. Interestingly, high doses of NO donors are suppressive in the expression of adhesion molecules in endothelial cells. In contrast, lower physiological concentrations induce immune activation, and the effects are likely associated with the regulation of adhesion molecules in an NFκB-dependent pathway [15]. These pieces of evidence indicate that the dose of NO may determine whether NO is protumorigenic or antitumorigenic. Expression of inducible nitric oxide synthase (iNOS) protein from myeloid-derived suppression cells (MDSCs) interferes with CD8^+^ T cell-mediated anti-tumor response [16]. In contrast, iNOS-expressing M1 macrophages inhibits tumor progression via high level NO and secretion of pro-inflammatory cytokines [17]. Endogenous NO, produced by nitric oxide synthase, regulates tumor progression via the immune system in a dose-dependent fashion [18]. Fewer studies have been performed on the effects of exogenous NO on immune cells in TME. Delivery of nitric oxide with a nanocarrier inhibits tumor progression through vessel normalization and increase of infiltrated CD8^+^ T cells in the TME [19]. The polynitrosated polyesters-based NO nanogenerator (NanoNO) enhances immunogenic cell death and potentiates cancer immunotherapy to prevent metastasis [20].

Several nitric oxide-releasing drugs have been used for a long time to treat cardiovascular diseases, such as isosorbide mononitrate (ISMN), which is a long-term nitric oxide-releasing drug administered to patients with hypertension [21]. Patients need to take ISMN daily to control blood pressure [22]. Moreover, other nitric oxide-releasing drugs, such as nitroglycerin and SNP, are commonly used as short-acting anti-angina agents to quickly decrease blood pressure [23, 24]. Although these drugs are used in millions of patients every year, the effects of nitric oxide-releasing drugs on cancer progression and the immune system are less studied.

Given that the direct effects of nitric oxide-releasing drugs on tumor cells or endothelial cells have been studied in vivo, we aimed to investigate whether exogenous nitric oxide regulates tumor progression through immune regulation. In this report, we studied the effects of several nitric oxide-releasing compounds (including FDA-approved drugs) on tumor progression in immune-competent mouse models. Furthermore, cytokine analysis and single-cell RNA sequencing were used to analyze various populations of immune cells and transcriptome alterations after treatment with exogenous nitric oxide-releasing drugs.

Our results demonstrated that low-dose exogenous nitric oxide induced an antitumor effect via CD8^+^ T cells. Additionally, low-dose nitric oxide increased a subset of Arg1-positive macrophages expressing M1-related genes, which may be associated with the antitumor immune response. Finally, a combination of low-dose nitric oxide releasing drugs and cisplatin increased the therapeutic effect in two different tumor-bearing mouse models.

## Materials and methods

### Cell culture reagents

B16F1 murine melanoma cells, LL2 murine Lewis lung carcinoma cells and CT26 murine colon carcinoma cells were used in this study. The B16F1, LL2 and CT26 cell lines were a kind gift from Dr. Ming-Shi Chang, Dr. Chao-Liang Wu and Dr. Liang-Yi Hung, respectively. These cell lines have been validated by American Type Culture Collection (ATCC). B16F1, LL2 and CT26 cells were cultured in high glucose Dulbecco’s modified Eagle medium (HG-DMEM) (Cat. No. SH30003.02; HyClone, Logan, UT, USA), low glucose Dulbecco’s modified Eagle medium (LG-DMEM) (Cat. No. SH30002.02; HyClone, Logan, UT, USA), and RPMI-1640 (Cat. No. CC110-0500; GeneDireX, Taiwan), respectively. S-Nitroso-N-acetyl-DL-penicillamine (SNAP) (N3398-50MG) and sodium nitroprusside (SNP) (BP453) were purchased from Sigma‒Aldrich (Darmstadt, Germany). Isosorbide-5-mononitrate (ISMN; Cat. No. HY-B0642) was purchased from MedChemExpress (NJ, USA). SNP and ISMN were dissolved in phosphate buffered saline (PBS) (10 mg/ml) and diluted in PBS for animal experiments. SNAP was dissolved in dimethyl sulfoxide (DMSO; Cat. No. 34943-2.5L; Honeywell-Fluka, Charlotte, NC, USA) and diluted in PBS. Briefly, 1 mg SNAP was dissolved in 100 μl DMSO and diluted with 900 μl PBS (1 mg/ml SNAP containing 10% DMSO as a stock concentration). SNAP (0.0004 mg/ml) containing 0.004% DMSO was used for 0.004 mg/kg SNAP treatment in animal experiments. The same concentration of DMSO-containing PBS was used as a control in the SNAP experiment in vivo. All media were supplemented with 10% fetal bovine serum (FBS, Cat. No. A6806-31, NQBB International Biological Corporation, Hong Kong), 100 U/ml penicillin, and 100 mg/ml streptomycin (Cat. No. SV30010; HyClone). Cells were maintained at 37 °C in a 5% CO_2_ incubator.

### Mouse tumor models

Six- to ten-week-old mice (C57BL/6, NOD-SCID and BALB/c) were obtained from the Laboratory Animal Center at National Cheng Kung University (Tainan, Taiwan). All study protocols involving mice were approved by the Animal Welfare Committee at National Cheng Kung University. To observe the effect of nitric oxide donors on tumor development, the mice were subcutaneously implanted with 2 x 10^5^ LL2, B16F1, or CT26 cells in the right flank. LL2 and B16F1 tumor-bearing mice were treated on Days 11, 12, 13, 16, 17, 18, 21, 22, and 23 with a nitric oxide donor by intraperitoneal injection (i.p.) after tumor cell implantation. The schedule involved injection for 3 consecutive days followed by no treatment for two days. CT26 tumor-bearing mice were treated with a nitric oxide donor starting at Day 8 after tumor implantation. The tumors were examined every 2-4 days by measuring the length (L) and width (W). The tumor volume (V) was calculated as V = (width x width x length x 0.52). Mice were sacrificed using CO_2_ when the tumor volume exceeded 2500 mm^3^.

### Total serum nitrite/nitrate measurement

Total serum nitrite and nitrate were measured using an OxiSelect In Vitro Nitric Oxide (Nitrite/Nitrate) Assay kit (STA-802, Cell BioLabs, San Diego, CA, U.S.A). Blood was collected in a tube without anticoagulant coating by submandibular blood collection 5 min after NO drug injection, and the serum was isolated by centrifugation at 2000x g for 10 min. Before the total nitrite/nitrate measurement, the serum was filtered with a 10-kDa MWCO ultrafilter through centrifugation at 5000x g for 10 min to reduce interference from protein. Fifty microliters of serum was cocultured with nitrate reductase for 1 hour at room temperature to recover nitrate into nitrite, and the total nitrite was detected with Griess reagents as a colored azo dye product. The absorbance was measured at 570 nm using a microplate reader (Multiskan FC Microplate Photometer, Thermo Fisher Scientific, Waltham, MA, USA).

### Single-cell suspension preparation

Splenocytes and tumor masses were isolated from LL2 tumor-bearing mice 20 days after tumor cell implantation. Tumor tissues were cut into 2-mm pieces and digested using collagenase A (1 mg/ml) (Cat. No: 10103586001; Roche, Basel, Switzerland) and DNase (100 U/ml) (Cat. No: 11284932001; Roche), and the cell suspensions were placed in a 37 °C shaker (150 rpm) for 60 min. Splenocytes were crushed directly into cell suspensions using wire mesh. The cell suspensions were treated with red blood cell lysis buffer on ice for 5 min to remove RBCs. After RBC lysis, the cell suspensions were washed twice with 2 ml of flow staining buffer (Cat. No: 554656; BD Pharmingen, San Diego, CA, U.S. and filtered through a 0.35-μm cell strainer (BD Pharmingen).

### CD326^+^ tumor cell and intratumoral CD8^+^ T lymphocyte isolation

A 1 x 10^7^ single-cell suspension of tumor tissue from LL2 tumor-bearing mice was used for CD326^+^ tumor cell and CD8^+^ T-cell isolation. Briefly, CD326^+^ cells were collected using a CD326-positive selection magnetic bead kit (Cat. No. 130-105-958; Miltenyi Biotec, Bergisch Gladbach, Germany) following the manufacturer’s instructions. CD326-positive tumor cells were used for the analysis of surface calreticulin, and CD326-negative cells were further used for CD8^+^ T-cell isolation with a CD8a-negative selection magnetic bead kit (Cat. No. 130-104-075; Miltenyi Biotec). The purity of the enriched CD8a^+^ T cells was homogeneously lysed in TRIzol for total RNA isolation.

### RNA extraction and quantitative real-time polymerase chain reaction (RT‒qPCR)

Total RNA was extracted from tumor-infiltrating CD8^+^ T cells, which were isolated using a CD8a^+^ T-cell isolation kit using TRIzol (Cyrusbioscience, Taiwan). MMLV reverse transcriptase (Promega, Madison, WI, USA) was used for cDNA synthesis. RT‒qPCR was performed with a StepOne system (Applied Biosystems, Foster City, CA, USA) and Fast SYBR-Green Master Mix (Thermo Fisher Scientific). Relative RNA expression was calculated using the 2^-ΔΔ^Ct method and normalized to glyceraldehyde 3-phosphate dehydrogenase (GAPDH) expression. The following forward and reverse primers for beta-catenin (Ctnnb1), transcription factor 7 (Tcf7), and GAPDH were used for RT‒qPCR: Ctnnb1 forward, GTT CGC CTT CAT TAT GGA CTG CC; Ctnnb1 reverse, ATA GCA CCC TGT TCC CGC AAA G; Tcf7 forward, CCT GCG GAT ATA GAC AGC ACT TC; Tcf7 reverse, TGT CCA GGT ACA CCA GAT CCC A; GAPDH forward, CAT CAC TGC CAC CCA GAA GAC TG; and GAPDH reverse, ATG CCA GTG AGC TTC CCG TTC AG. GAPDH served as an internal control.

### Flow cytometry

To investigate the alterations in infiltrating immune cells in the tumor microenvironment, we collected a single-cell suspension of tumor tissue from LL2 tumor-bearing mice. All infiltrating immune cells were stained with Fc blocker (Cat. No. 553141; BD Pharmingen) for 15 min on ice to reduce the nonspecific binding of antibodies on cells. Infiltrating CD8^+^ T cells were stained with BV510 rat anti-mouse CD45 (Cat. No. 563891; BD Pharmingen) and APC rat anti-mouse CD8a (Cat. No: 553035; BD Pharmingen). Mature dendritic cells in the tumor microenvironment were stained with BV510 rat anti-mouse CD45, BV421 hamster anti-mouse CD11c (Cat. No. 562782; BD Pharmingen), and APC rat anti-mouse CD86 (Cat. No. 561964; BD Pharmingen). To identify the population of TAM1-1 in the tumor, BV510 rat anti-mouse CD45, Alexa-488 rat anti-mouse IL7R (Cat. No. 561533; BD Pharmingen), and PE rat anti-mouse CD80 (Cat. No. 561955; BD Pharmingen) were used for surface staining; BV421 rat anti-mouse CD68 (Cat. No. 566389; BD Pharmingen), and PE-Cy7-conjugated Arg1 (Cat. No. 25-3697-82; eBioscience, Waltham, MA, USA) were used for intracellular staining via BD Cytofix/Cytoperm™ Plus Fixation/Permeabilization Kit (Cat. No. 555028; BD Pharmingen) according to the manufacturer’s instructions.

To determine the change in Th2 cells in splenocytes after low-dose SNAP treatment, splenocytes were collected at Day 20 after LL2 tumor implantation. Th2 cells were stained with Fc block for 15 min on ice and identified using PerCP-Cy5.5 rat anti-mouse CD45 (Cat. No. 550994; BD Pharmingen), APC rat anti-mouse CD4 (Cat. No. 552051; BD Pharmingen), and FITC rat anti-mouse IL-4 (Cat. No. 11-7042-82; eBioscienceTM). To measure the surface calreticulin in CD326^+^ tumor cells, CD326^+^ cells were isolated from a single-cell suspension of tumor tissue and stained with rabbit anti-mouse calreticulin (Ab2907; Abcam, Cambridge, UK) for 30 min on ice and further stained with goat anti-rabbit Alexa-488 (Ab150077; Abcam) for 30 min on ice. A Beckman CytoFLEX instrument was used for flow cytometry.

### Single-cell isolation and library preparation

Tumor tissues from LL2 tumor-bearing mice were cut into 2-mm pieces, and tumor tissues were prepared into a single-cell suspension with collagenase A (1 mg/kg) and type I DNase (100 U/ml). Next, red blood cells were removed with red blood cell lysis buffer, and the single-cell suspensions were resuspended in flow staining buffer. Finally, the cells were treated with an Fc blocker to reduce the nonspecific binding of the Fc receptor and antibodies. The cells were labeled with BD^TM^ Abseq Ab-logos (CD4 and CD8; Cat. No: 940108 and Cat. No: 940345), BD^TM^ sample tag (Cat. No: 633793), and rat anti-mouse BV510-CD45 and 7-AAD (Cat. No: 51-65875X; BD Pharmingen). A total of 5 x 10^5^ live immune cells (CD45 positive and 7-AAD negative) were sorted and resuspended in sample buffer for single-cell capture. Live CD45^+^ immune cells were captured using a BD Rhapsody^TM^ system and lysed. Briefly, cells (10,000 cells/group) were loaded into a cartridge, which had 200,000 wells for cell loading. Then, the cell-capture beads, which carried three different single-stranded DNA fragments to bind the DNA fragments of the sample tag, Abseq Ab-logos, and mRNA, were loaded into the well for cell binding. Nonbinding beads were removed from cartridge after washing with buffer. The cells captured by one bead were lysed, and mRNA was converted into cDNA by a Rhapsody^TM^ cDNA Kit (Cat. No. 633773; BD Pharmingen). Three libraries were established: a sample tag library, an Abseq library, and a whole transcriptome analysis (WTA) library. Briefly, a sample tag library was used to distinguish different samples. The Abseq library could easily identify specific immune cells for transcriptome analysis. The WTA library was used for gene expression analysis. Library construction was performed by the Molecular Diagnostics Group of the Department of Pathology, National Cheng Kung University Hospital, and the single-cell library was analyzed using the NovaSeq 6000 system by the Institute of Molecular and Genomic Medicine of the National Health Research Institute.

### Bioinformatics and computational biology analyses

Raw read data were aligned to the mouse genome (GRCm38.p6, gencode M19), and the expression matrix and sample tag matrix were produced by Rhapsody WTA pipeline V1.9. The control group with 4538 cells and the low-dose SNAP group with 4853 cells were used for further analysis. The count matrix was analyzed by the “Seurat” package [25] (Version 4.0.5). Genes and cells were removed under two conditions: cells with less than 700 feature genes and one feature gene with expression in less than 100 cells. After cell removal, we obtained 6563 cells (control: 3387, low-dose SNAP: 3176) for further analysis. The Seurat function “normalize Data” was used to normalize the raw count data. Variable genes were identified using the “FindVariableFeature” function. Default parameters were used for the Seurat function. Clusters were identified using the “FindClusters” function, and the resolution was 0.5. The dimensions of the data were reduced through UMAP. The differentially expressed genes in each cluster were identified via the “FindMarkers” function. When needed, clusters were combined into one cluster using the “Subset” function. Biological process (BP) and Kyoto Encyclopedia of Genes and Genomes (KEGG) analyses were performed using the “ClusterProfiler” package. Additionally, gene set enrichment analysis (GSEA) was performed using the “fgsea” package in R. We selected two GSEA databases for our analysis: hallmark gene sets and ontology gene sets. Moreover, MetaCore software was used for pathway analysis of significant genes. The cluster annotations were established based on signature genes that were approved by previous studies [26, 27]. Highly expressed genes in CD8^+^ T cells were assessed using f tests, t tests, and *p* values < 0.05. A list of significantly differentially expressed genes was imported into Metacore software to construct signaling pathways. The significance cutoff point for enrichment of a pathway was a *p* value < 0.05.

### Cytokine array

The spleen was isolated from LL2 tumor-bearing mice on the 20th day after tumor implantation. A single-cell suspension of splenocytes was prepared as described previously. To stimulate the cytokines secretion, splenocytes (5 x 10^5^) were cultured with LL2 lysate in 2 ml of RPMI 1640 medium at 37 °C for 24 h. The culture medium was collected by centrifugation at 1500 rpm for 5 min, and the supernatant was collected and stored at -80 °C until use. Mouse Cytokine Array Panel A (Cat. No. ARY006; R&D Systems, Minneapolis, MN, USA) was used to measure the levels of 40 cytokines. For cytokine analysis, a 1-ml sample was mixed with 15 μl of reconstituted Mouse Cytokine Array Panel A Detection Antibody Cocktail for 1 hour at room temperature, and the mixture was added to the array membrane and incubated overnight at 4 °C. Then, the array membranes were incubated with 2 ml of streptavidin-HRP for 30 min at room temperature. The array membranes were exposed to X-ray film, and the expression levels of each cytokine were quantitated by ImageJ software (Development by Dr. Wayne Rasband; National Institutes of Health).

### Depletion of CD8^+^ T cells

The T-cell depletion protocol was performed as previously described with minor modifications. Anti-CD8 (clone: 53-6.7, Cat. No. BE0004-1, Bio X Cell, Lebanon, NH, U.S. A) (200 μg per mouse) or rat IgG2a isotype control antibody (clone: 2A3, Cat. No. BE0089; Bio X Cell) was intraperitoneally injected into mice on Days 10 and 15. Approximately 90% of CD8^+^ T cells were depleted, as determined by flow cytometry analysis.

### Biotin switch assay of S-nitrosylation protein and western blotting

To measure nitric oxide-induced protein nitrosylation, THP-1 cells were treated with different doses of SNP for 30 min, and 1 mg/ml protein was used for detection of S-nitrosylation protein. Here, S-nitrosylation of proteins was performed using an S-nitrosylated protein detection assay kit based on the biotin switch method (1006518, Cayman) following the manufacturer’s instructions. Total biotinylated proteins were purified with streptavidin-agarose beads (Thermo Fisher Scientific) for 1 hour at room temperature. Purified proteins were detected by western blotting. Nonpurified proteins were used as the input control, and the level of total nitrosylation protein was measured.

The following antibodies were used for western blotting: anti-GAPDH (Cat. No. GTX100118; GeneTex, Hsinchu, Taiwan) and anti-Hsp90 (Cat. No. 610418; BD Pharmingen). Total nitrosylation proteins were detected using an avidin-HRP conjugated antibody. Nitrosylated proteins were detected by western blotting following standard protocols. Images were obtained utilizing a BioSpectrum AC imaging system (UVP, CA, USA) following the manufacturer’s instructions.

### Immunohistochemistry

Tumor-bearing mice were sacrificed 2 days after the second treatment (20th day after the implantation of tumor cells), and tumor tissues were collected in optimal cutting temperature (OCT) compound. Tumor tissues were sliced at a thickness of 5 μm. The tissue was fixed with 3.7% paraformaldehyde, and endogenous peroxidase was inactivated with 3% H_2_O_2_ at room temperature. Angiogenesis was detected with anti-CD31 (1:200 dilution) (Clone: MEC13.3, Cat. No. 550274, BD Pharmingen) antibody. The level of angiogenesis was assessed at a magnification of 100 ×. Five randomly chosen fields per sample from each mouse were evaluated.

### Statistical analysis

All statistical analyses were performed using GraphPad Prism 8. All statistical analyses of tumors were performed with two-way ANOVA. Statistical analysis of the percentage of immune cell populations derived from FACS analysis was performed with t tests. A *p* value < 0.05 was considered statistically significant.

## Results

### Low-dose nitric oxide donors induced an antitumor response in immune-competent tumor-bearing mouse models but not in immune-deficient mice

**Fig. 1:**
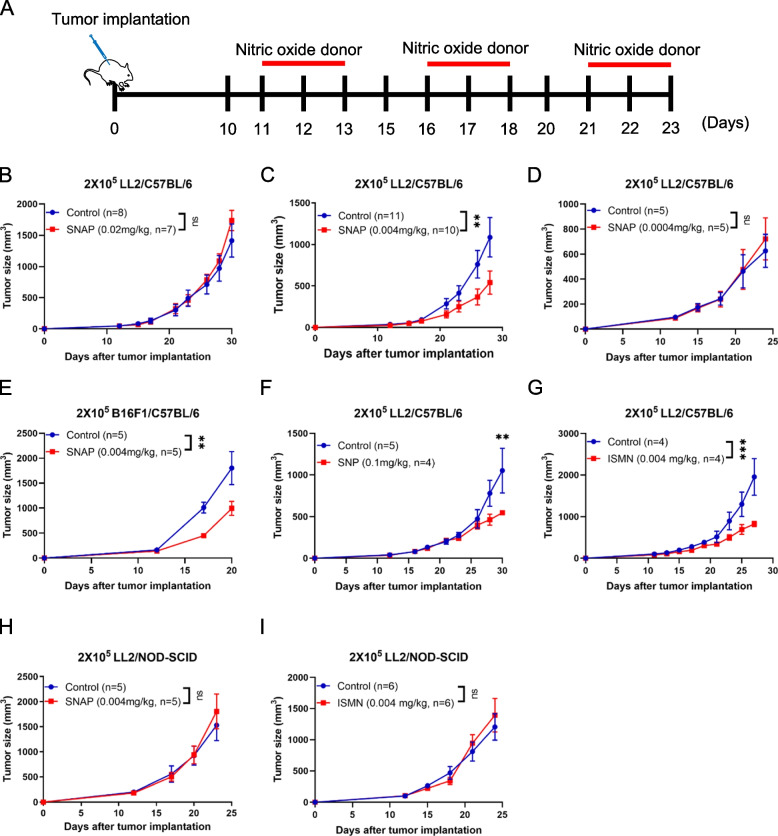
Low doses of nitric oxide donors induce an antitumor response that is immune dependent. (**A**) Schematic of the tumor-bearing mouse model and the timeline of treatment with the nitric oxide donor S-nitroso-N-acetyl-DL-penicillamine (SNAP). LL2 tumor-bearing C57BL/6 mice received (**B**) 0.02, (**C**) 0.004, and (**D**) 0.0004 mg/kg SNAP. (**E**) B16F1 tumor-bearing C57BL/6 mice were administered 0.004 mg/kg SNAP by intraperitoneal injection. (**F**) LL2 tumor-bearing mice were administered SNP (0.1 mg/kg). (**G**) LL2 tumor-bearing C57BL/6 mice were administered 0.004 mg/kg isosorbide mononitrate (ISMN). (**H**) LL2 tumor-bearing immunodeficient NOD-SCID mice were administered 0.004 mg/kg SNAP or (I) 0.004 mg/kg ISMN by intraperitoneal injection. In all mice, 2 × 10^5^ Lewis lung carcinoma LL2 cells or B16F1 melanoma cells were implanted in the right flank. Tumor volumes were measured every 2-4 days beginning on the twelfth day after tumor implantation. The tumor results are presented as the means ± SEMs. *p < 0.05, **p < 0.01, ***p < 0.001. ns, no significant difference. All *p*-values were obtained by two-way ANOVA

To investigate the effect of the NO donor S-nitroso-N-acetyl-DL-penicillamine (SNAP) on tumorigenesis in a mouse model, the murine Lewis lung carcinoma (LL2) tumor cell line was subcutaneously implanted in the right flank of C57BL/6 mice. Tumor-bearing mice were treated with a nitric oxide donor at various doses, and the treatment schedule is illustrated in Fig. 1A. High-dose SNAP (0.02 mg/kg) did not affect tumor progression in LL2 tumor-bearing mice (Fig. 1B). However, a low dose of SNAP (0.004 mg/kg) induced an antitumor response in LL2 tumor-bearing mice (Fig. 1C). The antitumor effect of low-dose SNAP was dose dependent. A 10-fold lower dose of SNAP (0.0004 mg/kg) did not affect tumor growth in LL2 tumor-bearing mice (Fig. 1D). In addition, low-dose SNAP treatment (0.004 mg/kg) also induced an antitumor response in the B16F1 melanoma orthotropic mouse model (Fig. 1E). The FDA-approved nitric oxide drug sodium nitroprusside (SNP) reduced tumor growth in the 0.1 mg/kg treatment group (Fig. 1F). Similarly, another FDA-approved nitric oxide donor drug, isosorbide-5-mononitrate (ISMN), inhibited tumor growth at the same low dose (0.004 mg/kg) in an LL2 tumor-bearing animal model (Fig. 1G). The results of three different nitric oxide donors indicated that a low dose of exogenous nitric oxide inhibited tumor growth. We then determined whether a low dose of nitric oxide donor increased NO levels or nitrosylated proteins in vivo and in vitro. The concentration of NO in mouse serum was increased 5 min after 0.1 mg/kg SNP treatment (*p* value = 0.09 in Supplementary Fig. 1 A). Due to an increase in NO levels after treatment with the low-dose NO-donating drug SNP in vivo, we further tested whether low-dose SNP affected protein nitrosylation in vitro. Therefore, THP-1 monocytic cells were treated with increasing doses of SNP, and protein nitrosylation was determined with an S-nitrosylated protein detection kit based on the biotin-switch method [28]. SNP enhanced levels of total nitrosylated protein in THP-1 cells even at the very low concentration of 1 μM (Supplementary Fig. 1 B). Additionally, low-dose SNP also enhanced the nitrosylation of heat-chock protein 90 (Hsp90), which is a known target of S-nitrosylation by NO [29] (Supplementary Fig. 1 C).

We next examined whether immune modulation was essential in mediating the antitumor response induced by low-dose SNAP and ISMN. LL2 tumor cells were implanted in the right flank of immune-deficient mice (NOD-SCID), and the therapeutic effects of low-dose SNAP and ISMN (0.004 mg/kg) were monitored. Low-dose SNAP and ISMN (0.004 mg/kg) did not affect tumor growth (Fig. 1H and I). Therefore, the immune system was essential for the low-dose SNAP-mediated antitumor response.

### Low-dose SNAP regulated splenic cytokines and the Th2 cell population

Systemic immune activation is important for the response to immunotherapy [30], and we determined whether cytokines were regulated by low-dose SNAP treatment. Cytokine expression levels were analyzed using a cytokine array. We isolated tumor tissues and splenocytes on Day 20 after tumor implantation. Tumor weight and tumor size were reduced (Supplementary Fig. 2 A) in the low-dose SNAP (0.004 mg/kg) treatment group. To stimulate cytokine secretion, we cocultured LL2 cell lysates with splenocytes for 24 hours, and the supernatants were collected for cytokine detection using a cytokine array. The cytokines IL-6 and IL-10 are important for Th2-mediated function in cancer progression and exhibited reduced levels in the low-dose SNAP treatment group. Additionally, the levels of the cytokines IFN-γ and TNF-α were higher in the low-dose SNAP treatment group compared with in the control group (Supplementary Fig. 2 B-D). To further determine whether Th2 cells were decreased by SNAP, splenocytes were collected from LL2 tumor-bearing mice at Day 20 after SNAP treatment. The proportion of Th2 cells, which may be associated with B cells, decreased after low-dose SNAP (0.004 mg/kg) treatment (Supplementary Fig. 2 E and F). Our results suggested that low-dose SNAP (0.004 mg/kg) influenced the Th2 T-cell population, which is associated with alterations in cytokines in the spleen.

### Single-cell RNA sequencing determines the landscape of the TME in response to low-dose SNAP treatment

**Fig. 2:**
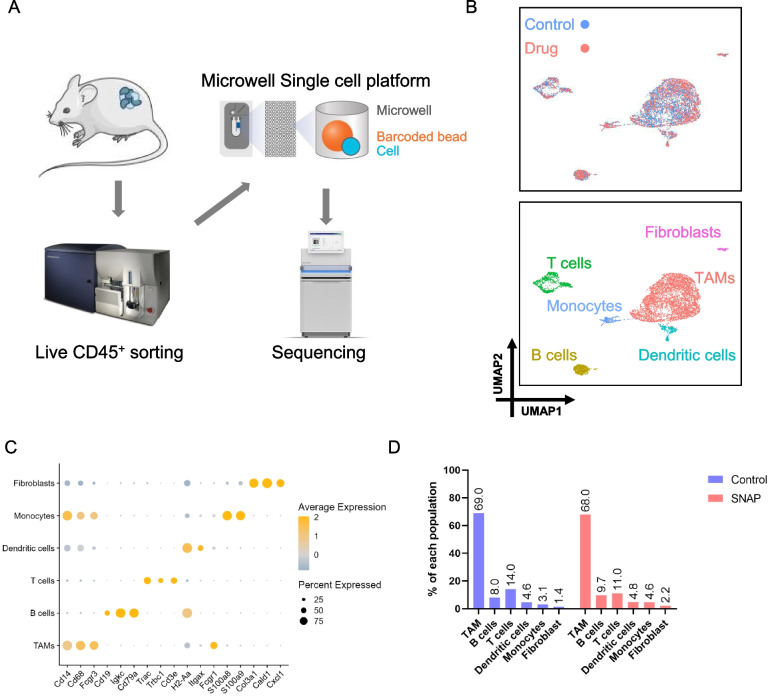
The top 2 abundant infiltrating immune cells were tumor-associated macrophages and T cells. (**A**) Schematic of the study design. Single-cell RNA sequencing was used to analyze infiltrating immune cells isolated from tumor-bearing mice. (**B**) Clustering of intratumoral CD45^+^ cells and visualization of the subset. Uniform manifold approximation and projection (UMAP) of single-cell RNA sequencing data from 6563 cells (Control: 3387; Drug: 3176; *upper*) revealed six clusters determined by 14 specific markers (Supplementary Fig. 3). Each dot plot represents one cell. (**C**) Bubble heatmap determining the expression levels of specific marker genes in the CD45^+^ subsets and fibroblasts. (**D**) The percentages of CD45^+^ subsets and fibroblasts in the control and low-dose SNAP treatment groups

To further understand the complex changes in immune cells in the tumor microenvironment, we used single-cell RNA sequencing based on a microwell-based system (BD Rhapsody) to determine this profile. The changes in T cells and macrophages can be best detected by single-cell RNA sequencing given that these cell types are the most abundant immune cells in the tumor microenvironment. Tumor tissues were collected from LL2 tumor-bearing mice of the SNAP treatment group and control group and were processed into a single-cell suspension. Single-cell suspensions from three mice in each group were mixed for further analysis. Tumor-infiltrating immune cells (CD45^+^ and 7-AAD^-^) were isolated from LL2 tumor-bearing mice of the SNAP treatment group (0.004 mg/kg) and control group (PBS containing 0.004% DMSO). CD45^+^ cells were stained with the BD sample tag, which carries a specific sequence, to identify the different groups during sequencing (Fig. 2A). A total of 9391 cells were analyzed in the two groups (4538 cells for the control group and 4853 cells for the low-dose SNAP group).

First, we classified cells into 11 clusters through clustering resolution selection (Supplementary Fig. 3 A). To identify cluster specificity, we categorized the marker genes and displayed the top five differentially expressed genes (DEGs) in each cluster (Supplementary Fig. 3 B and Supplementary Data 1). We further analyzed the gene expression intensities of 14 common markers to determine the immune cell type in these 11 clusters (Supplementary Fig. 3 C). Finally, we annotated each cluster and reduced the 11 clusters to 6 cell types (Fig. 2B) according to the intensity of the marker genes: tumor-associated macrophages (Clusters 0, 1, 2, and 3; Cd14^+^ and Cd68^+^), B cells (Cluster 4; *Igkc*^+^, and *Cd79a*^+^), T cells (Clusters 5, 8, and 9; *Cd3e*^+^ and *Trac*^+^), dendritic cells (Cluster 7; *H2-Ab1*^+^ and *Itgax*^+^), monocytes (Cluster 7; CD14^+^, *S100a8*^+^, *S100a9*^+^, and *Fcgr1*^-^) [31], and fibroblasts (Cluster 10; *Cald1*^+^ and *Col3a1*^+^). The expression levels of marker genes in the cluster were determined (Fig. 2C and Supplementary Fig. 3 D). We further analyzed the composition of CD45-positive cells between the control group and the low-dose SNAP treatment group. Tumor-associated macrophages (TAMs) were the major competent intratumoral immune cells in the two groups (69% and 68%). In addition, approximately 11-14% of cells were T cells (Fig. 2D). Our single-cell RNA sequencing results indicate that TAMs are the most abundant intratumoral immune cells. In addition, low-dose SNAP treatment did not remarkably alter the ratio of macrophage and T-cell populations in tumor microenvironments.

### Low-dose SNAP treatment increases CD8^+^ T cells and natural killer (NK) cells

**Fig. 3:**
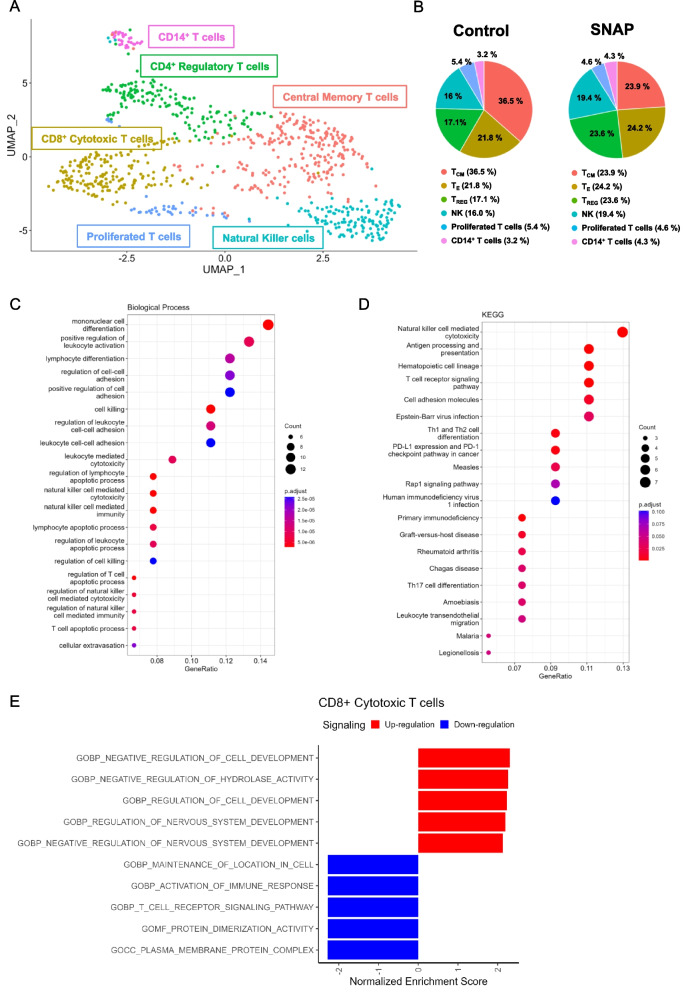
Low-dose SNAP increases CD8^+^ effector T cells, and the cytotoxicity pathway is enriched in these cells. (**A**) Subclustering of T lymphocytes and visualization of the subsets. Dimension reduction by UMAP displaying six subsets of T lymphocytes (814 cells). (**B**) Distribution of each subset of T lymphocytes in the control and SNAP treatment groups. (**C**) Biological process and (**D**) KEGG pathway analyses of 92 DEGs (avg_log_2_-fold change > 0.5) in CD8^+^ T cells. The GeneRatio indicates the number of significant genes associated with the Gene Ontology (GO) term divided by the total number of genes in the related pathway in the database. (**E**) MSigDB ontology gene set analysis of 1117 differentially expressed genes (DEGs) in Cluster 1 CD8^+^ cytotoxic T cells after low-dose SNAP treatment; the list of DEGs is shown in the Supplementary Data. 3. DEGs were identified using the “FindMarker” function in the Seurat package. The red bar represents the upregulated pathway after low-dose SNAP treatment. All pathways were statistically significant (*p*-value < 0.05). The *p*-value of GSEA was calculated by permutation

Given that CD8^+^ T cells are one of the key immune cells responsible for antitumor immunity, we first aimed to study the effect of low-dose SNAP treatment on the T cell population. To efficiently identify CD4^+^ and CD8^+^ cells, we used CD4 and CD8 Ab-seq antibodies, which carry the antibody and a specific barcode for single-cell RNA analysis. To determine the populations of T cells, we subclustered the T lymphocytes that were identified by *CD3e* and *Trac* and used uniform manifold approximation and projection (UMAP) for dimension reduction (Supplementary Fig. 4 A). A total of six clusters were identified. There were two CD8^+^ T-cell clusters: Cluster 0, central memory T cells (*Tcf7*, and *S1pr1*) and Cluster 1, CD8^+^ cytotoxic T cells (*Cd8a*, CD8 (Ab), *Gzmb*, *Nkg7*, and *Prf1*); one CD4^+^ T-cell cluster: Cluster 2, CD4^+^ regulatory T cells (CD4 (Ab), *Ctla4*, *Il2ra*); two CD3e^+^ T cells: Cluster 4, proliferating T cells (*Mki67*, *Top2a*, *Pclaf*, *Stmn1*) and Cluster 5, CD14^+^ T cells; and Cluster 3, natural killer cells (*CD3e*^-^, *Gzma*, *Nkg7*, *Prg1*, and *Gzmb*) (Fig. 3A and Supplementary Figure 4B and C and Supplementary Data 2). Notably, during low-dose SNAP treatment, the population of Cluster 1 CD8^+^ cytotoxic T cells (Effector T cells; T_E_) increased from 21.8% to 24.2%, and Cluster 3 natural killer (NK) cells increased from 16% to 19%. In contrast, central memory T cells (T_CM_) in Cluster 0 were significantly decreased from 36.5% to 23.9% (Fig. 3B). Analysis of the top 5 expressed genes in T_CM_ identified Cd69 (Supplementary Data 2); therefore, Cd69 and Tcf7 were analyzed in 5 T cell populations and NK cells. Coexpression of Tcf7 and Cd69 was observed only in T_CM_ (Supplementary Fig. 5A), closely matching the exhausted T cell progenitor 1 (Tex^prog1^) populations reported recently [32]. Additionally, biological process analysis of upregulated genes in Cluster 1 CD8^+^ cytotoxic T cells (avg_log_2_FC > 0.5) indicated that several gene groups were enriched in leukocyte activation and cytotoxicity (Fig. 3C). Moreover, KEGG pathway analysis suggested that the upregulated genes were related to the NK cell-mediated immune response (e.g., *Gzmb*, *Prf1*, *Klrd1*, *Klrk1*, *Itgal*, and *Itgb2*) (Fig. 3D). To further investigate which mechanisms were altered in Cluster 1 CD8 cytotoxic T cells after low-dose SNAP treatment, 1117 DEGs derived from the low-dose SNAP group (Supplementary data 3) were used for gene set enrichment analysis (GSEA). GSEA results indicated that the DEGs of Cluster 1 CD8^+^ cytotoxic T cells exposed to low-dose SNAP treatment were enriched in negative regulation of hydrolase activity (Fig. 3E). GSEA results implied that hydrolase inhibition might correlate with the low-dose SNAP-induced antitumor response. Indeed, previous studies indicated that T cell activation resulted in a decrease in adipocyte triglyceride lipase, a triacylglycerol hydrolase [33]. These results suggested that low-dose SNAP increased the number of Cluster 1 CD8^+^ cytotoxic T cells and that this cluster exhibited an expression pattern associated with potentially increased cytotoxicity. GSEA results also suggest that low-dose SNAP treatment might regulate hydrolase activity to enhance T cell activation. On the other hand, GSEA analysis of 937 DEGs in NK cells indicated cluster 5 NK cells contained the interferon-gamma response gene signature (Supplementary Fig. 5B and supplementary data 4).

### CD8^+^ T cell-mediated immunity is required for tumor inhibition by low-dose nitric oxide donors

**Fig. 4:**
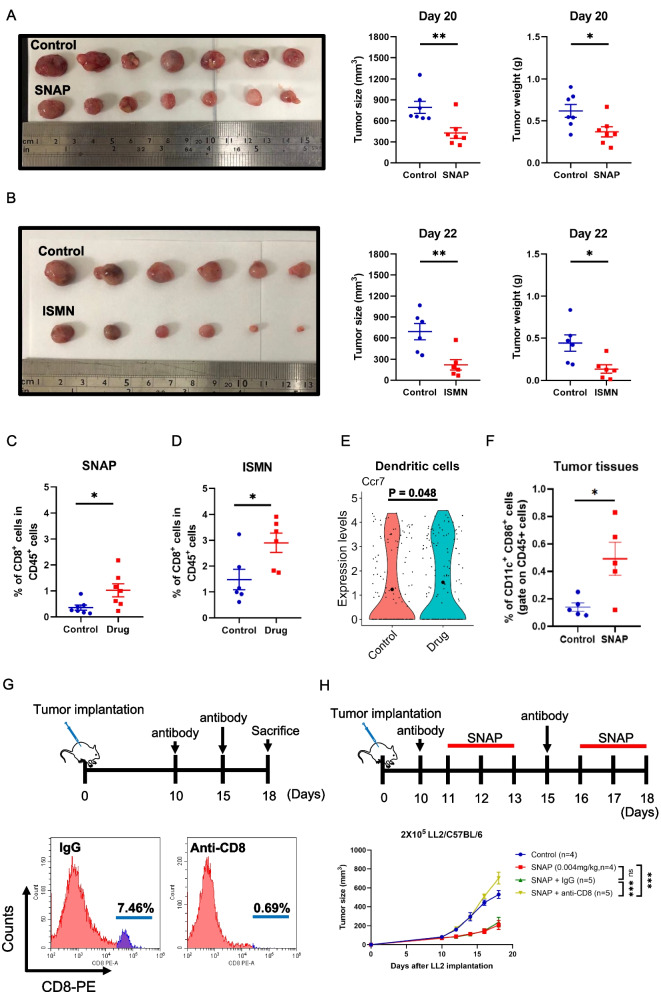
CD8^+^ T cells are essential for the repression of tumor growth mediated by low-dose SNAP. (**A**) Tumor tissues were isolated from LL2 tumor-bearing mice after SNAP treatment *(left)*, and the tumor sizes *(middle)* and the tumor weights *(right)* were analyzed on the 20^th^ day. (**B**) Tumor tissues were isolated from LL2 tumor-bearing mice after ISMN treatment *(left)*, and the tumor sizes *(middle)* and the tumor weights *(right)* were analyzed on the 20^th^ day. (**C**) FACS analysis of intratumoral CD8^+^ T cells between the control group and low-dose SNAP treatment group. (**D**) FACS analysis of intratumoral CD8^+^ T cells between the control group and the low-dose ISMN treatment group. The mice were sacrificed, and tumor-infiltrating lymphocytes were analyzed on the 20^th^ day after implantation of LL2 tumor cells. (**E**) Violin plot revealing Ccr7 expression levels in dendritic cells. Each dot represents one cell. (**F**) Flow cytometry analysis of intratumoral mature dendritic cells between the control group and low-dose SNAP treatment group. (**G**) Schematic of CD8^+^ T-cell depletion in mice (*upper*). Representative histogram of CD8^+^ expression on splenocytes (*lower*). (**H**) Simplified diagram of CD8^+^ T-cell depletion in the LL2 tumor-bearing mice (*upper*). LL2 tumor-bearing mice were treated with low-dose SNAP (0.004 mg/kg) and anti-CD8 antibody (200 μg/time) (*lower*). Anti-IgG antibody was used as the control. Both antibodies and SNAP were administered via intraperitoneal injection. The column scatter dot plot represents the mean values ± SEMs. *p < 0.05, **p < 0.01, versus the control group. *P*-values of the tumor results and violin plots were obtained by two-way ANOVA and the Wilcox test, ***p < 0.001, ns, no significant difference. The tumor volumes were measured every 2 days beginning on the 10^th^ day after tumor implantation

Immunosuppressive cytokines, such as IL-6 and IL-10, interfere with the antitumor effects of CD8^+^ and CD4^+^ T cells in the tumor microenvironment [34, 35]. Low-dose SNAP (0.004 mg/kg) treatment decreased IL-6 and IL-10 secretion by splenocytes (Supplementary Fig. 2E). Moreover, single-cell RNA sequencing analysis indicated that low-dose SNAP treatment altered CD8^+^ T cells with activation gene signatures. Therefore, we further analyzed the percentages of CD8^+^ T cells in the tumor microenvironment after low-dose SNAP treatment using flow cytometry. Mice were treated with low-dose SNAP (0.004 mg/kg) and sacrificed on the 20^th^ day after LL2 tumor implantation. The low-dose SNAP group exhibited smaller tumor sizes and weights than the control group (Fig. 4A). In addition, another NO donor, ISMN, also reduced tumor volume and weight upon low-dose treatment (0.004 mg/kg) (Fig. 4B). Infiltrating CD8^+^ T cells were increased by low-dose SNAP or ISMN (Fig. 4C and D). These results demonstrated that an increase in CD8^+^ T cells may play a role in the antitumor effect induced by SNAP or ISMN treatment.

To investigate the potential mechanisms by which low-dose SNAP regulates CD8^+^ T cell activation, we collected the expression profiles in the subset of T lymphocytes (Clusters 0, 1, 2, 4, and 5), excluding Cluster 3 NK cells. We further gated CD8^+^ T cells (*n* = 302) with Ab-seq antibodies (CD8 (Ab) and CD4 (Ab)) using SeqGeq software, a platform for single-cell analysis (Supplementary Fig. 6A). In addition, we selected 338 significant genes among 8369 genes in CD8^+^ T cells (average expression > 0, *p* value < 0.05, percentage of expressing cells > 10, SNAP/Control fold change >1.5; Supplementary Data 5) for pathway analysis by Metacore software. Alterations in gene expression in CD8^+^ T cells were highly related to the Wnt/beta-catenin pathway (Supplementary Fig. 6B), which was recently reported to regulate CD8^+^ T-cell activation [36]. Therefore, we further isolated intratumoral CD8^+^ T cells by magnetic beads and measured the mRNA levels of beta-catenin-related genes by quantitative real-time polymerase chain reaction (RT‒qPCR). Beta-catenin (Ctnnb1) was significantly downregulated, and its targeted transcriptional factor 7 (Tcf7) was slightly downregulated upon treatment with low-dose SNAP (Supplementary Fig. 6C and D). In addition to the Wnt/beta-catenin pathway, cross-presentation by mature dendritic cells also contributes to CD8^+^ T cell activation [37], and the expression of C-C chemokine receptor 7 (Ccr7) in conventional dendritic cells (cDCs) plays an important role in delivering intact tumor antigen to tumor-draining lymph nodes [38] and is also a marker in the maturation of dendritic cells [39]. Single-cell RNA sequencing data revealed that *Ccr7* expression levels in dendritic cells were increased after low-dose SNAP treatment (Fig 4E). Therefore, we next examined whether low-dose SNAP increased the number of mature and activated dendritic cells in the tumor microenvironment. Flow cytometry analysis indicated that the number of cells double-positive for the dendritic cell marker CD11c and maturation marker CD86 was increased after low-dose SNAP treatment (Fig 4F). A previous study indicated that nitric oxide treatment induced immunogenic cell death (ICD) to control tumor progression [20]. ICD is a process in which dying cells release damage-associated molecular patterns (DAMPs) to activate the maturation of dendritic cells and induce CD8^+^ T cell activation to kill cancer cells [40]. To investigate whether the low-dose SNAP-mediated antitumor effect was correlated with ICD, we isolated tumor cells from LL2 tumor-bearing mice through CD326 magnetic beads and measured levels of the ICD marker, surface calreticulin. However, surface calreticulin was only weakly enhanced by low-dose SNAP treatment (Supplementary Fig. 7). These results demonstrated that low-dose SNAP treatment increased dendritic cell maturation and CD8^+^ T cells in the tumor microenvironment. To further determine whether CD8^+^ T cells play an essential role in the antitumor response, CD8^+^ T cells were depleted with an anti-CD8 antibody. Two doses of anti-CD8 antibody depleted approximately 90% of CD8^+^ T cells, as demonstrated by flow cytometry (Fig. 4G). The low-dose SNAP-mediated antitumor response was almost abolished by CD8^+^ T cell depletion (Fig. 4H). These results demonstrated that CD8^+^ T cells were required for the antitumor effects induced by low-dose SNAP.

The results above indicated that the low-dose SNAP-mediated antitumor response was dependent on CD8^+^ T cells and correlated with mature dendritic cells. In addition, SNAP treatment influenced the Wnt/beta-catenin pathway of CD8^+^ T cells, which may be related to CD8^+^ T-cell activation.

### Low-dose SNAP increased a TAM subtype that coexpressed M1- and M2-related markers

**Fig. 5:**
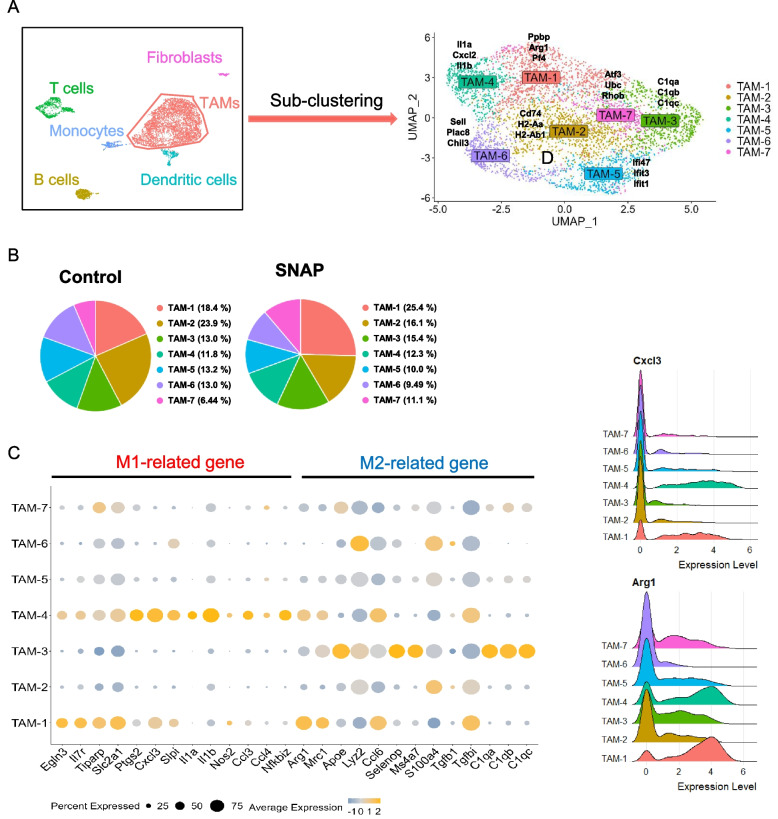
Low-dose SNAP treatment induces alterations in macrophage subsets. (**A**) Subclustering of tumor-associated macrophages (TAMs) by UMAP and identifying the top three DEGs in each subset of TAMs. The top three DEGs were identified using the “FindAllMarker” function in the Seurat package. (**B**) The distribution of each TAM defined by gene expression patterns in the control or low-dose SNAP treatment groups. (**C**) The expression levels of M1-related and M2-related genes in the TAM subset. (**D**) Ridge plots determining the expression distributions of M1-related genes (Cxcl3) and M2-related genes (Arg1) in the TAM subsets. The X-axis represents log-normalized expression levels

NO is an important molecule for macrophage differentiation [41]; therefore, low-dose NO donor SNAP treatment may regulate TAM polarization. To further investigate whether low-dose SNAP treatment modulated TAM differentiation, we analyzed alterations in the expression levels of M1 and M2 macrophage markers. TAMs were subclustered into 7 clusters based on the following markers: TAM1, *Ppbp*, *Arg1*, and *Pf4*; TAM2, *Cd74*, *H2-Aa*, and *H2-Ab1*; TAM3, *C1qa*, *C1qb*, and *C1qc*; TAM4, *Il1a*, *Cxcl2*, and *Il1b*; TAM5, *Ifit47*, *Ifit3*, and *Ifit1*; TAM6, *Sell*, *Plac8*, and *Chil3*; and TAM7, *Atf3*, *Ubc*, and *Rhob* (Fig. 5A). TAM1 expressed M2 markers Arg1 and Mrc1. TAM2 expressed major histocompatibility complex 2 (MHC-II) and macrophage migration inhibitory factor (MIF) receptor CD74. TAM3 expressed the complement component 1 complex genes *C1qa*, *C1qb*, and *C1qc* TAM4 expressed the proinflammatory genes *Il1a*, *Il1b*, and *Ptgs2*. TAM5 expressed the interferon-stimulated genes *Rsad2*, *Isg15*, and *Ifit1*. TAM6 expressed *Plac8* and *Chil3*. TAM7 expressed *Klf2* and *Egr1* (Supplementary Fig. 8).

To investigate the effect of low-dose SNAP on the TAM subtype, we calculated the proportion of each TAM subset. Low-dose SNAP dramatically altered the amounts of two TAM subsets, TAM1 and TAM2 (Fig. 5B). TAM1 was increased from 18.4% to 25.4% after low-dose SNAP treatment, whereas TAM2 was decreased from 23.9% to 16.1%. Given that M1 and M2 macrophages frequently exert opposite effects on tumor progression, we analyzed M1-related genes and M2-related genes in the TAM subtypes [42–48]. TAM4 cells expressed more M1-related genes (*Ccl3*, *Nos2*, and *Il1b*), and TAM3 cells exhibited higher mRNA expression of M2-related genes (*Apoe*, *C1qa*, *C1qb*, and *C1qc*). However, TAM2 cells exhibited reduced mRNA expression levels of M1- and M2-related genes (Fig. 5C). Of note, low-dose SNAP treatment increased a particular subtype of macrophages (TAM1) that coexpressed M1- and M2-related genes, such as *Cxcl3* and *Arg1* (Fig. 5D), suggesting that low-dose SNAP regulated the inflammatory response in TAMs.

### Coexpression of the M1-like gene expression signature and Arg1 in a subpopulation of macrophages induced by SNAP

**Fig. 6:**
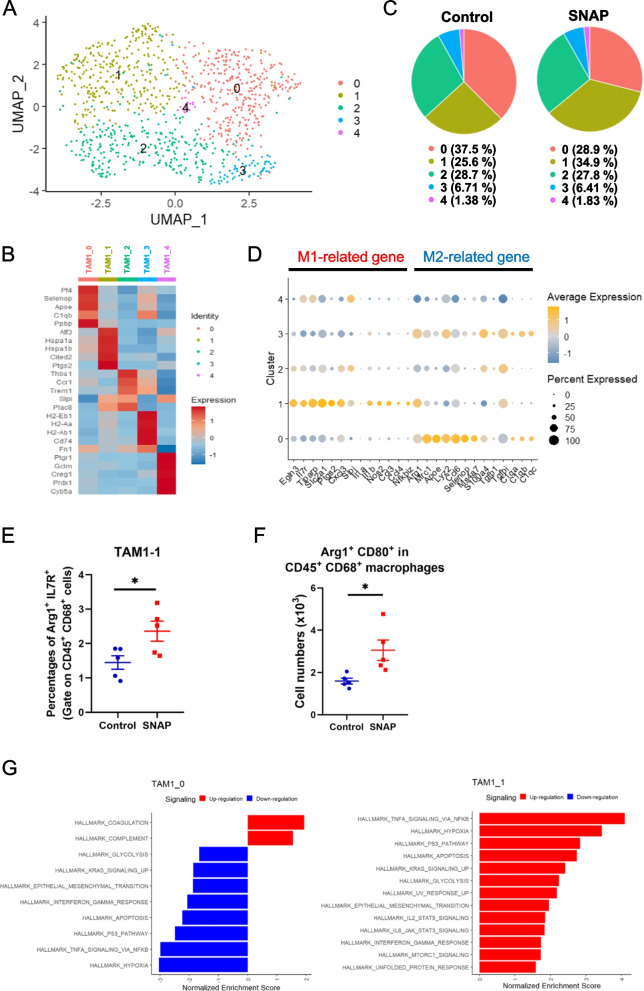
Low-dose SNAP induces a subset of macrophages displaying an M1-related gene expression signature and Arg1 expression. (**A**) Subclustering of TAM1 (978 cells) and visualization of the subsets. UMAP embedding of single-cell RNA sequencing data revealed five clusters using the “FindCluster” function. Each dot represents one cell. (**B**) Average expression levels of the top 5 DEGs in each subset of TAM1 cells. (**C**) Distribution of each TAM1 subset in the control and SNAP treatment groups. (**D**) Bubble heatmap showing the expression levels of M1-related genes and M2-related genes in the subset of TAM1 cells. (**E**) Flow cytometry analysis of intratumoral TAM1-1 cells and (**F**) CD80^+^ Arg1^+^ macrophages between the control and low-dose SNAP treatment groups. (**G**) MSigDB hallmark gene set enrichment assay (GSEA) analysis of DEGs in the TAM1-0 (*left*) and TAM1-1 (*right*) clusters. TAM1-0 and TAM1-1 DEGs were determined using the “FindMarker” function in the Seurat package and are displayed in Supplementary Data 6. All pathways were significant (*p*-value < 0.05). Tumor tissues were collected Day 20 after tumor implantation and used for flow cytometry analysis

Arg1-expressing M2 macrophages in general play an immunosuppressive role through the consumption of extracellular L-arginine [49]; however, CD206-expressing M2 macrophages can be converted into cells that enhance adaptive and innate antitumor immune responses [50]. Our single-cell analysis revealed that low-dose SNAP increased the proportion of TAM1 cells that coexpressed M1- and M2-related genes (Fig. 5C). In addition, TAM1 cells did not express IL-10 (Supplementary Fig. 9), which is a key cytokine associated with immunosuppression. Therefore, it is important to further characterize the unique macrophage subpopulation induced by low-dose SNAP. We subclustered TAM1 cells, and 5 subsets were identified (TAM1-0 to TAM1-4) (Fig. 6A). The top 5 averaged genes in each subtype of TAM1 were calculated and visualized in a heatmap (Fig. 6B). TAM1-0 cells expressed *Pf4 (Cxcl4)* and *Selenop*, which induced M2 macrophage differentiation [51], and TAM1-1 cells had high expression of heat shock protein family A members (*Hspa1a* and *Hspa1b*) and prostaglandin-endoperoxide synthase 2 (Ptgs2). TAM1-2 cells expressed higher levels of thrombospondin 1 (Thbs1). TAM1-3 cells expressed MHC-class II molecules (*H2-Eb1*, *H2-Aa*, and *H2-Ab*), and TAM1-4 cells exhibited increased prostaglandin reductase 1 (Ptgr1) expression. Notably, TAM1-1 cells had lower *Mrc1* (CD206) expression levels and higher expression levels of *Ptgs2*, a gene involved in the inflammatory response in M1 macrophages [52]. Low-dose SNAP treatment reduced the TAM1-0 population but increased the TAM1-1 population. On the other hand, the TAM1-2, TAM1-3, and TAM1-4 populations were not affected by SNAP (Fig. 6C). To further determine whether M1-related genes were expressed in Arg1-expressing macrophages, we analyzed M1- and M2-related genes in subsets of TAM1 cells. TAM1-0 cells expressed more M2 macrophage-related genes, and TAM1-1 cells exhibited increased expression of M1 macrophage-related genes (Fig. 6D). Indeed, flow cytometry analysis also indicated that low-dose SNAP treatment increased the population of Arg1^+^ IL7R^+^ macrophages and Arg1^+^ CD80^+^ macrophages (Fig. 6E and F). These data suggested that low-dose SNAP treatment decreased M2-like macrophages (TAM 1-0). In addition, a unique subset of Arg1-expressing macrophages (TAM1-1) that highly expressed M1-related genes was significantly increased after low-dose SNAP treatment.

To further understand the potential roles of TAM1-0 and TAM1-1 in the tumor microenvironment, DEGs were used (800 genes in TAM1-0 cells, 991 genes in TAM1-1 cells) for hallmark gene set enrichment analysis (GSEA) (Supplementary Data 6). TAM1-0 cells had enhanced levels of coagulation and complement pathways and reduced levels of glycolysis, hypoxia, and TNF-α signaling and IFN-γ response pathways. TAM1-1 cells exhibited opposite trends based on GSEA. TAM1-1 cells displayed enrichment in proinflammatory response pathways, including TNF-α signaling, the IFN-γ response, and IL-6-JAK-STAT3 signaling (Fig. 6G). Based on GSEA, TAM1-0 cells may function as protumor macrophages; on the other hand, TAM1-1 macrophages may possess antitumor properties. Therefore, the antitumor response of low-dose SNAP was correlated with a decrease in protumor macrophages and an increase in a unique set of Arg1-positive macrophages with an antitumor gene expression signature.

### Low-dose SNAP enhanced the therapeutic effect of cisplatin in tumor-bearing mouse models

**Fig. 7:**
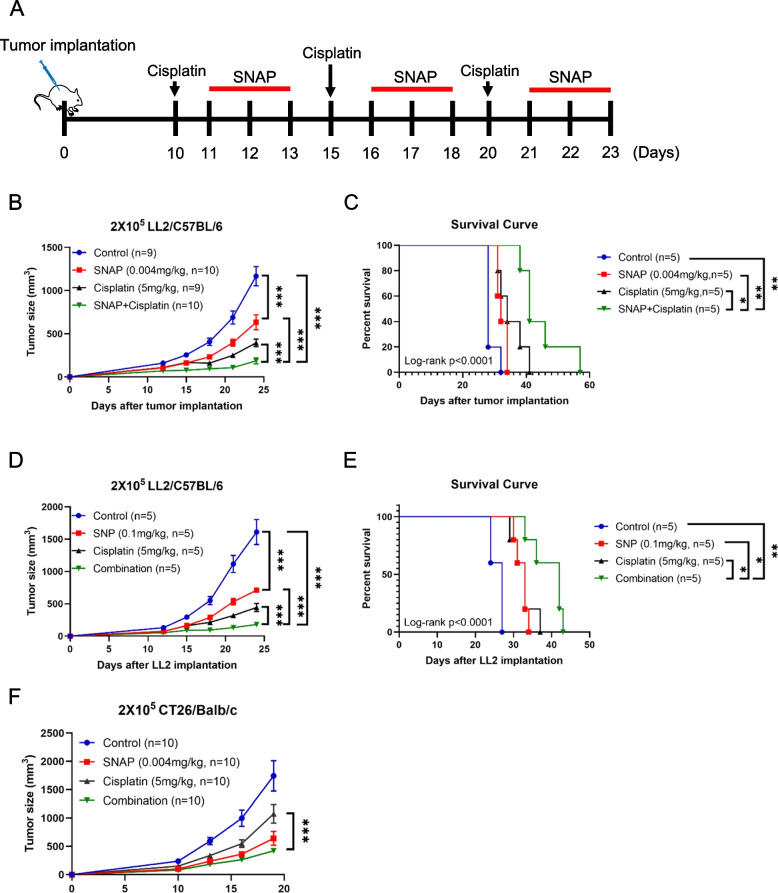
Combination of nitric oxide donors and cisplatin induces an additive therapeutic effect in vivo. (**A**) Schematic representation of the combination treatment schedule of cisplatin and SNAP. (**B**) LL2 tumor-bearing mice were administered cisplatin (5 mg/kg), SNAP (0.004 mg/kg) or both, and tumor growth was measured. (**C**) The Kaplan–Meier survival curve of mice administered combination treatment with low-dose SNAP and cisplatin. (**D**) LL2 tumor-bearing mice were administered cisplatin (5 mg/kg), SNP (0.1 mg/kg) or both, and tumor growth was measured. (**E**) The Kaplan–Meier survival curve of the mice administered combination treatment with low-dose SNP and cisplatin. (**F**) CT26 tumor-bearing mice were treated with cisplatin (5 mg/kg), SNAP (0.004 mg/kg) or both, and tumor growth was measured. The *p*-values of the tumor growth curve were obtained by two-way ANOVA, *p < 0.05, **p < 0.01, ***p < 0.001. The log-rank *p*-values of the survival curve were obtained using the log-rank (Mantel‒Cox) test. The tumor volumes were measured every 2-4 days after tumor implantation

Finally, combination treatment with chemotherapeutic drugs and immunomodulation agents is frequently employed in cancer patients to prolong their survival [53]. For example, immune checkpoint blockade plus cisplatin has been used to treat NSCLC [54]. Our results indicated that low-dose SNAP inhibited tumor growth via regulation of the immune system; therefore, we further tested the concept that low-dose SNAP treatment might enhance the therapeutic effect of the chemotherapeutic drug cisplatin. Mice were treated with low-dose SNAP (0.004 mg/kg) and/or cisplatin (5 mg/kg), and tumor growth was measured. The combination treatment protocol is illustrated in Fig. 7A. In the LL2 tumor-bearing mouse model, a combination of low-dose SNAP and cisplatin enhanced the cancer therapeutic effect and prolonged survival compared with cisplatin or low-dose SNAP alone (Fig. 7B and C). Similarly, a low dose of SNP, another nitric oxide donor, enhanced the therapeutic effect and extended the survival of tumor-bearing mice (Fig. 7D and E). To further investigate whether the effective combination therapy effect can be observed in another mouse strain, BALB/C mice were implanted with CT26 colon tumor cells and treated with the same doses of SNAP, cisplatin, or both. Increased antitumor effects were observed in the CT26 tumor-bearing mouse model upon combination treatment with low-dose SNAP and cisplatin (Fig. 7F). These results indicated that low-dose SNAP enhanced the antitumor efficacy of a common chemotherapeutic drug, cisplatin. To investigate the potential mechanism responsible for the enhancement of therapeutic effects in the combination study, we performed single-cell RNA sequencing on live tumor-infiltrating immune cells from four groups (control, low-dose SNAP, cisplatin, and combination). A total of 6 clusters (TAMs, monocytes, dendritic cells, B cells, T cells and fibroblasts) were identified based on DEGs and 14 common markers (Supplementary Fig. 10A and B). T cells and NK cells were clustered into 5 clusters based on DEGs: CD3^+^ CD4^-^ CD8^-^ double negative T cells, CD4 regulatory T cells (*Cd4*, *Il2ra*, and *Foxp3*), CD8 cytotoxic T cells (*Cd8*, *Gzmb*, and *Ifng*), proliferating T cells (*Mki67*, *Top2a*, and *Pclaf*), and NK cells (*Cd3e* negative, *Klrk1*, *Nkg7*, and *Gzma*), and alterations in these clusters were assessed among the four groups of mice (Supplementary Fig. 10C). Combination treatment with low-dose SNAP and cisplatin increased the number of NK-annotated cells based on gene expression patterns. Similar percentages of CD8^+^ cytotoxic T cells were observed between the cisplatin group and the combination group. However, interestingly, combination treatment inhibited the androgen response in CD8^+^ cytotoxic T cells in GSEA (Supplementary Fig. 10D), which may inhibit CD8^+^ T-cell function [55]. These results demonstrated that an increase in NK cells is associated with an enhanced therapeutic effect in combination treatment. Additionally, the additive effect of combination treatment might correlate with the downregulation of the androgen response in CD8^+^ T cells.

## Discussion

IN this report, we first identified a dose-dependent effect of three different nitric oxide donors on tumor progression in subcutaneous lung tumor animal models (LL2 cells in C57BL/6 mice) and orthotopic melanoma animal models (B16F10 cells in C57BL/6 mice). The antitumor effect of a low-dose nitric oxide donor was also observed in another syngeneic immunocompetent animal model (CT-26 cells in BALB/c mice).

To investigate the immune mechanism responsible for the antitumor effect of low-dose nitric oxide donors, we used three different approaches: (1) systemic splenic cytokine expression, (2) single-cell RNA sequencing of tumor microenvironment immune cells, and (3) flow cytometry analysis of immune cells. A cytokine array revealed that IL-6 and IL-10, which are important for Th2-mediated functions, were decreased by low-dose nitric oxide donors. On the other hand, IFN-γ and TNF-α levels were increased after treatment. Flow cytometry confirmed the decrease in Th2 cells. The tumor microenvironment in the LL2 mouse model consists mainly of macrophages (approximately 70%) and T cells (approximately 10%) in our single-cell RNA analysis. We first investigated whether T cells are associated with antitumor effects. Single-cell RNA sequencing revealed that low-dose nitric oxide donors increased the population of CD8^+^ cytotoxic T cells and natural killer (NK) cells but decreased central memory T cells (T_CM_). The increase in infiltrating CD8^+^ T cells was further verified by flow cytometry. In addition, the SNAP-mediated antitumor response was decreased after depletion of CD8^+^ T cells, demonstrating the essential role of CD8^+^ T cells. Moreover, pathway analysis of CD8^+^ T cells demonstrated that low-dose SNAP treatment altered the Wnt/beta-catenin pathway, which is upstream of Tcf. This finding suggests that low-dose SNAP-regulated T-cell activation may be related to alterations in the Wnt/beta-catenin pathway. Consistent with our findings, a previous report indicated that nitric oxide-releasing derivatives of oleanolic acid inhibit Wnt/beta-catenin signaling in colon cancer [56].

Macrophages of the TME were initially clustered into seven expression types (TAM1 to TAM7) in our immune-competent mice (Fig. 5). TAM1 and TAM4 expressed M1-related genes, suggesting antitumor characteristics. However, cluster TAM1 macrophages also expressed M2-related genes, including arginase 1. Therefore, we subclustered TAM1 into 5 subclusters: TAM1-0 to TAM1-4 macrophages. Further GSEA revealed that TAM1-0 may represent M2 protumor macrophages, whereas TAM1-1 may function as M1 antitumor macrophages. The decrease in TAM1-0 macrophages and the increase in TAM1-1 macrophages may contribute to the regression of tumors by NO donors. At present, we are unable to determine the influence of a specific subset of macrophages due to the lack of a specific antibody against the targeting population of macrophages. On the other hand, the subset of Arg1-expressing macrophages (TAM1-0) exhibited high ApoE expression. ApoE expression in M2 macrophages regulates tumor migration in gastric cancer [57]. Additionally, human *APOE* levels were elevated in pancreatic ductal adenocarcinoma and correlated with patient survival; notably, *APOE*^-/-^ mice exhibited an increase in infiltrating CD8^+^ T cells [58]. Whether this subset of Arg1-expressing macrophages plays an important role in the tumor microenvironment warrants further study in the future.

Nitric oxide-releasing drugs have been used to treat several diseases, especially cardiovascular diseases. Our results indicated that 0.004 mg/kg SNAP ($1.81\times {10}^{-6} \mathrm{M}\times 10\frac{\mu l}{1}\mathrm{g})$ treatment or the FDA-approved drug ISMN inhibited tumor progression. It has been reported that 10^-8^ M to 10^-4^ M SNAP was sufficient to induce carotid arterial relaxation [59], implying that a lower dose of SNAP exerts a physiological effect. Indeed, 0.004 mg/kg SNAP slightly decreased systolic blood pressure 5-15 min after intraperitoneal injection. Therefore, it is necessary to monitor the patient’s cardiovascular function when the low-dose releasing drugs are used in treating cancer in the future (data not shown). In addition, increasing nitrite levels from 140 to 220 nM could significantly decrease blood pressure in humans [60]. Additionally, nitric oxide levels greater than 100 nM easily activate the downstream molecule soluble guanylyl cyclase (sGC) [61]. The physiological NO concentration is suggested to range from 100 pM (or below) to approximately 5 nM [62]. Altogether, the concentrations of SNAP and ISMN used in this study are probably within the physiological range.

Interestingly, higher concentrations of NO did not exhibit antitumor effects (Fig 1). Given that the roles of NO are multifaceted and dose dependent, we hypothesized that one of the important contributing factors is angiogenesis. Therefore, we investigated whether low-dose and high-dose SNAP had different effects on angiogenesis in the tumor microenvironment using CD31, an angiogenesis marker. Immunohistochemistry staining of the tumor microenvironment revealed a lower number of CD31-positive cells in the 0.004 mg/kg treatment group compared with the control group. In contrast, CD31 staining in the tumor microenvironment was not altered by 0.02 mg/kg SNAP treatment (Supplementary Fig. 11A and B). Given that angiogenesis may cause immune suppression, a decrease in angiogenesis may further contribute to immune activation induced by low-dose exogenous nitric oxide [63].

Low-dose SNAP treatment reduced the Th2 population and the cytokines IL-6 and IL-10 in splenocytes. Moreover, low-dose SNAP treatment increased the expression levels of the Th1 cytokines IFN-γ and TNF-α, suggesting that low-dose SNAP treatment not only regulated immune cells in the TME but also systemically enhanced proinflammatory cytokines. Indeed, a low dose of the nitric oxide donor NOC-18 increased the number of murine Th1 cells [64]. On the other hand, IL-6 is also secreted by Th2 cells. High IL-6 levels correlate with a worse prognosis in patients with prostate cancer [65]. These results suggest that low-dose SNAP may also regulate systemic immunity to control tumor progression, especially by maintaining the balance between Th1 and Th2 cells.

Our cytokine assay on spleen cells indicated that several cytokines were altered by nitric oxide. The altered expressions of IL-6 and IL-10 are the most prominent. On the other hand, decreases in CCL3, CCL4, and CXCL12 were also observed. CXCL12 activates CXCR4 and CXCR7 chemokine receptors, and the signal axis is dysregulated in multiple types of cancer. Targeting the CXCL12 signal axis is a promising therapy for cancer. The expression of CXCL12 was decreased by a low dose of nitric oxide, which may in part contribute to the anti-tumor effects observed in our animal study [66]. CCL3 (MIP-1α) and CCL4 (MIP-1β) are located in chromosome 17. The expression of CCL3 and CCL4 is increased in chronic lymphocytic leukemia, and can be used as biomarkers in the therapy targeting chronic lymphocytic leukemia [67, 68]. However, the role of CCL3 and CCL4 appears to exert both antitumor and pro-tumor behavior which is context dependent in solid cancers [69].

Interestingly, low-dose SNAP treatment also increased regulatory T cells. Regulatory T cells play an important role in self-tolerance. In addition, regulatory T cells also inhibit the function of effector T cells and antigen-presenting cells [70]. Anti-CTLA4 or anti-PD-1 antibodies have been reported to enhance the antitumor response through reduction of regulatory T cell activity on effector T cells. In this regard, a combination of low-dose SNAP and anti-CTLA4 antibodies may induce a syngeneic effect on tumor regression.

Recently, cocktail treatment has become a popular approach for cancer patients, especially a combination of immune therapy and chemotherapy. For example, pembrolizumab plus chemotherapy has been used to treat metastatic non-small cell lung cancer and resulted in a better prognosis than single-drug treatment [71]. Given that cisplatin is frequently used for lung cancer and melanoma chemotherapy, cisplatin was used as a proof-of-concept drug for combination treatment with nitric oxide donors. The present results demonstrated that the combination of low-dose SNAP and cisplatin treatment exhibited a better therapeutic effect than cisplatin treatment alone.

## Conclusions

In summary, low-dose SNAP treatment induced CD8^+^ T cell activation and regulated Arg1-expressing macrophages with antitumor properties in the tumor microenvironment. Additionally, low-dose SNAP treatment reduced the expression of the anti-inflammatory cytokines IL-10 and IL-6 in the spleen. Furthermore, combination low-dose SNAP and cisplatin treatment induced an additive antitumor effect. Taken together, low-dose nitric oxide donors may emerge as a promising strategy for improving cancer immunotherapy. To the best of our knowledge, this is the first study to demonstrate the antitumor effect of low-dose exogenous nitric oxide (no carrier) on the immune system. These findings might provide a new treatment strategy for cancer using nitric oxide-releasing drugs in cancer patients.

## Supplementary Information

**Additional file 1: Supplementary Figure 1.** Low-dose SNP treatment increased nitric oxide levels in vivo and induced protein nitrosylation in vitro.(A) Nitric oxide level in the serum of control or 0.1 mg/kg SNP-treated mice was determined by measuring total serum nitrite and nitrate levels. (B). Total nitrosylated protein levels in THP-1 cells were detected using anti-biotin antibody after 30 min of SNP treatment, and GAPDH levels served as the loading control in western blotting. (C) Nitrosylated Hsp90 (SNO-Hsp90) was measured by western blotting after streptavidin agarose bead collection. The column scatter dot plot represents the mean values ± SEMs. The *p*-value was obtained by t test. **Supplementary Figure 2.** Low-dose SNAP regulates cytokine expression levels and the Th2 cell population in the spleen. (A) The tumor weight and tumor size on the day mice were sacrificed. Tumor tissues were isolated from LL2 tumor-bearing mice. The mice were sacrificed on the 20^th^ day after LL2 tumor implantation. (B) A cytokine array was used to determine the cytokine levels in the culture medium of splenocytes, which were co-cultured with LL2 cell lysates at 37 °C for 24 hr. The splenocytes were collected from 0.004 mg/kg SNAP treated mice (*upper*) and PBS-containing 0.004 % DMSO treated mice (*lower*). (C) The coordinates of every cytokine in the array. (D) The expression levels of each cytokine in the control and low-dose SNAP treatment groups. (E) The quadrants represent the percentage of CD4^+^ and/or IL-4-positive cells. (F) The percentage of IL-4-positive cells in the CD4^+^ population (type 2 T helper cells; Th2). LL2 tumor-bearing mice were administered SNAP (0.004 mg/kg) at 11 to 13 and 16 to 18 days, and splenocytes were isolated and analyzed. The column scatter dot plot represents the mean values ± SEMs. *p < 0.05, versus the control group. ns, no significant difference. *P*-values of the Th2 percentages were obtained by t test. **Supplementary Figure 3.** Reduction of cell clusters from 11 to 6 by the levels of marker genes expressed in CD45^+^ cells. (A)Clustering of CD45^+^ and subset visualization. UMAP dimensionality reduction of total CD45^+^ cells (6563 cells) was executed based on visualization of relevant cell clusters. All clusters were determined using the “FindCluster” function in the Seurat package. (B) Heatmap displaying the top 5 differentially expressed genes (DEGs) and mRNA levels in 11 clusters, and the top five DEGs list is presented in Supplementary Data 1. (C) Feature plot depicting single-cell gene expression of marker genes. *Cd14*, *Cd68*, and *Itgam* are marker genes for tumor-associated macrophages (TAMs). Cd3e and Trac were used for T-cell identification. *S100a8*, *S100a9* and *Fcgr1* were used to identify monocytes (monocytes did not express *Fcgr1*). Dendritic cells were identified by *Itgax* (CD11c) and *H2-Ab1* (MHC II molecule). B cells expressed the *Igkc* and *Cd79a* genes. *Cald1* and *Col3a1* were expressed on fibroblasts. (D) Violin plot showing the expression levels of each marker gene in each cluster. **Supplementary Figure 4.** T lymphocytes are distinguished into 6 clusters and identified by marker genes. (A) Subclustering of T lymphocytes and visualization of the subsets.UMAP dimensionality reduction of 813 T lymphocytes (control: 463; drug: 350) was executed based on visualization of relevant cell clusters. All clusters were determined using the “FindCluster” function in the Seurat package. (B) Heatmap displaying the top 5 DEGs and mRNA levels in six subsets of T lymphocytes, and the gene list is shown in Supplementary Data 2. (C) Feature plot showing the single-cell gene expression of marker genes. Cd3e represents T cells. CD8a (Ab) and *Cd8a* are marker genes of CD8^+^ T cells. *Gzma*, *Gzmb*, *Prf1*, and *Nkg7* were used to identify the cytotoxic function of cells. *Tcf7* is an essential marker of central memory T cells. CD4 (Ab) was used to identify CD4^+^ T cells, and *Il2ra* (CD25) and Ctla4 determined the regulatory function of CD4^+^ T cells. *Mki67* is a proliferation marker.** Supplementary Figure 5.** TCF7 and CD69 expression levels in T-cell subsets and GSEA analysis of NK cells. (A) Violin plot showing *Tcf7* and *Cd69* expression levels in T-cell subsets. (B) MSigDB Hallmark gene set displayed significant overlap with the DEGs of natural killer cells. The DEGs of NK cells were identified by the “FindMarker” function in the Seurat package and the DEGs list was shown in supplementary data 4. *p*-values of the violin plot were obtained by ANOVA. **p < 0.01, ***p< 0.001, ****p< 0.0001 versus the TAM1 group. **Supplementary Figure 6.** Low-dose SNAP regulates the Wnt/beta-catenin pathway in CD8^+^ T cells. (A) T lymphocytes were identified by CD4 (Ab) and CD8 (Ab) Abseq antibodies. Q3 are designated as CD8^+^ T cells. (B) Bar chart showing the significant pathways in CD8^+^ T cells after low-dose SNAP treatment. Significant genes were selected for pathway analysis, and the gene list is shown in Supplementary Data 5 (*p*-value < 0.05, fold change > 1.5, percentages of expressed cells > 10, and the *p*-value was obtained by t test). (C) Ctnnb1 (beta-catenin) and (D) Tcf7 mRNA levels in intratumoral CD8^+^ T cells from the control and 0.004 mg/kg SNAP treatment groups as determined by RT‒qPCR. The column scatter dot plot represents the mean values ± SEMs. *p*-values of the column scatter dot plot were obtained by t test. *p< 0.05, ns, no significant difference.** Supplementary Figure 7.** Low-dose SNAP treatment did not significantly induce the expression of surface calreticulin in LL2 tumor-bearing mice. Flow cytometry analysis of surface calreticulin in CD326-positive tumor cells isolated from LL2 tumor-bearing mice between the control and 0.004 mg/kg SNAP treatment groups. The mice were sacrificed at Day 20 after LL2 cell implantation. The column scatter dot plot represents the mean values ± SEMs. The *p*-values were obtained by t tests. **Supplementary Figure 8.** The mRNA levels of the top 5 differentially expressed genes in the TAM subsets. Violin plot demonstrating the expression levels of the top five DEGs in each subset of TAMs. The Y-axis represents log-normalized expression levels. **Supplementary Figure 9.** TAM1 cells do not express the immunosuppressive cytokine IL-10. Ridge plots determining IL-10 expression in the TAM1 subset. The X-axis represents log-normalized expression levels. *p*-values of the violin plot were obtained using the Wilcoxontest. ns, no significant difference. **Supplementary Figure 10.** Single-cell RNA sequencing analysis of combination cisplatin and low-dose SNAP treatment. (A) Feature plot demonstrating the gene expression of marker genes. *Cd14*, *Cd68*, and *Itgam* are marker genes for tumor-associated macrophages (TAMs). *Cd3e* and *Trac* were used for T-cell identification. *S100a8*, *S100a9* and *Fcgr1* were used to identify monocytes (monocytes did not express *Fcgr1*). Dendritic cells were identified by *Itgax* (CD11c) and *H2-Ab1* (MHC II molecular). B cells expressed the genes *Igkc* and *Cd79a*. *Cald1* and *Col3a1* were expressed on fibroblasts. (B) *Left panel*: Clustering of intratumoral CD45^+^ cells and visualization of each type of cell. Uniform manifold approximation and projection (UMAP) of single-cell RNA sequencing data from 6690 cells (Control: 1909; Low-dose SNAP: 1943, Cisplatin: 1474, and Drug Combination: 1364) revealed six clusters determined by 14 specific markers (Supplementary Fig. 2). *Right panel*: Subclustering of T lymphocytes and visualization of the subsets. UMAP dimensionality reduction of 403 T lymphocytes was executed based on visualization of relevant cell clusters. (C) *Upper panel*: Violin plots revealed the expression levels of the marker genes for the subset of T cells. The Y-axis represents log-normalized expression levels. *Cd3e* serve as a T-cell marker; *Foxp3*, *Il2ra*, and *Cd4* serve as CD4 regulatory T-cell markers; *Gzmb*, *Ifng*, and *Cd8a* serve as CD8 cytotoxic T-cell markers; *Mki67*, *Top2a*, and *Pclaf* serve as proliferating T-cell markers; *Klrk*, *Nkg7*, and *Gzma* serve as NK cell markers. *Lower panel*: The percentages of each group of the subset of T cluster within total immune cells. (D) GSEA of CD8^+^ cytotoxic T cell DEGs between combination and cisplatin treatment. The red bar chart and blue bar chart show upregulated and downregulated pathways in the combination treatment group, respectively. **Supplementary Figure 11.** Treatment with 0.004 mg/kg but not 0.02 mg/kg SNAP decreased angiogenesis in the tumor microenvironment. (A) Frozen sections of 0.004 mg/kg or (B) 0.02 mg/kg SNAP-treated and control tumors from LL2 tumor-bearing mice stained with the angiogenesis marker CD31 (red). Five randomly chosen areas in each tumor sample were used to calculate the CD31^+^ area. The column scatter dot plot represents the mean values ± SEMs. *p < 0.05, ns, no significant difference. *p*-values of the column scatter dot plot were obtained by t test.

**Additional file 2.** Top 5 differentially expressed genes of intratumoral CD45^+^ cells.

**Additional file 3.** Top 5 differentially expressed genes in T lymphocyte subsets.

**Additional file 4.** Cluster 1 CD8^+^ cytotoxic T cell differentially expressed genes following low-dose SNAP treatment.

**Additional file 5.** Differentially expressed genes and entrezgene IDs for GSEA in cluster 3 NK cells.

**Additional file 6.** The expression profile of CD8a (Ab)-positive cells identified by SeqGeq platform.

**Additional file 7.** Differentially expressed genes of TAM1-0 and TAM1-1.

## Data Availability

All data generated or analyzed during this study are included in this published article. Single-cell RNA sequencing data has been uploaded to GEO.

## Abbreviations

DEGs: Differentially expressed genes
GSEA: Gene Set Enrichment Analysis
IFN-γ: Interferon gamma
ISMN: Isosorbide mononitrate
KEGG: Kyoto Encyclopedia of Genes and Genomes
LL2: Lewis lung carcinoma
M1: Type 1 macrophage
M2: Type 2 macrophage
NO: Nitric oxide
NSCLC: Non-small cell lung carcinoma
SNAP: S-Nitroso-N-acetyl-DL-penicillamine
SNP: Sodium nitroprusside
TAM: Tumor-associated macrophage
T_CM_: Central memory T cells
Th1: Type 1 helper T
Th2: Type 2 helper T
TME: Tumor microenvironment
TNF-α: Tumor necrosis factor alpha
T_REG_: Regulatory T
UMAP: Uniform manifold approximation and projection
