# Supplementary material for: Repurposing nitric oxide donating drugs in cancer therapy through immune modulation
Source: J Exp Clin Cancer Res. 2023 Jan 14;42:22. doi: 10.1186/s13046-022-02590-0 (PMC9840268; doi:10.1186/s13046-022-02590-0)
Supplement: Supplementary file 1 — Additional file 1: Supplementary Figure 1. Low-dose SNP treatment increased nitric oxide levels in vivo and induced protein nitrosylation in vitro.(A) Nitric oxide level in the serum of control or 0.1 mg/kg SNP-treated mice was determined by measuring total serum nitrite and nitrate levels. (B). Total nitrosylated protein levels in THP-1 cells were detected using anti-biotin antibody after 30 min of SNP treatment, and GAPDH levels served as the loading control in western blotting. (C) Nitrosylated Hsp90 (SNO-Hsp90) was measured by western blotting after streptavidin agarose bead collection. The column scatter dot plot represents the mean values ± SEMs. The p-value was obtained by t test. Supplementary Figure 2. Low-dose SNAP regulates cytokine expression levels and the Th2 cell population in the spleen. (A) The tumor weight and tumor size on the day mice were sacrificed. Tumor tissues were isolated from LL2 tumor-bearing mice. The mice were sacrificed on the 20th day after LL2 tumor implantation. (B) A cytokine array was used to determine the cytokine levels in the culture medium of splenocytes, which were co-cultured with LL2 cell lysates at 37 °C for 24 hr. The splenocytes were collected from 0.004 mg/kg SNAP treated mice (upper) and PBS-containing 0.004 % DMSO treated mice (lower). (C) The coordinates of every cytokine in the array. (D) The expression levels of each cytokine in the control and low-dose SNAP treatment groups. (E) The quadrants represent the percentage of CD4+ and/or IL-4-positive cells. (F) The percentage of IL-4-positive cells in the CD4+ population (type 2 T helper cells; Th2). LL2 tumor-bearing mice were administered SNAP (0.004 mg/kg) at 11 to 13 and 16 to 18 days, and splenocytes were isolated and analyzed. The column scatter dot plot represents the mean values ± SEMs. *p < 0.05, versus the control group. ns, no significant difference. P-values of the Th2 percentages were obtained by t test. Supplementary Figure 3. Reduction of cell clusters [file 13046_2022_2590_MOESM1_ESM.docx]

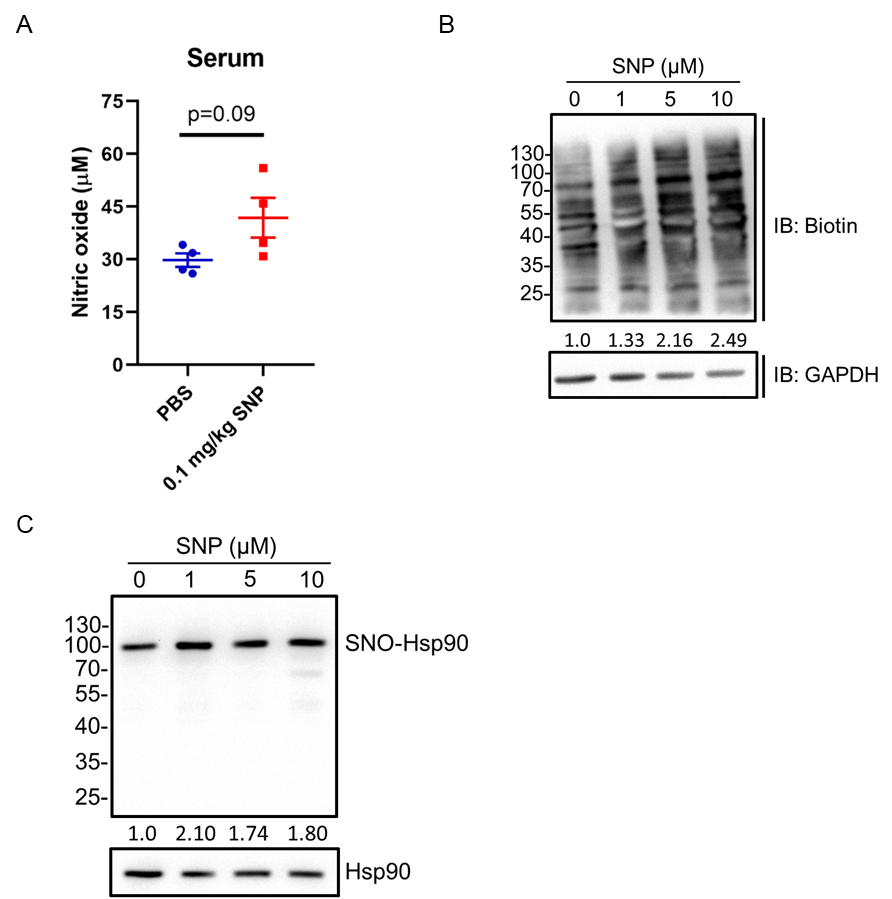


**Supplementary Figure 1: Low-dose SNP treatment increased nitric oxide levels in vivo and induced protein nitrosylation in vitro.**

(A) Nitric oxide level in the serum of control or 0.1 mg/kg SNP-treated mice was determined by measuring total serum nitrite and nitrate levels. (B). Total nitrosylated protein levels in THP-1 cells were detected using anti-biotin antibody after 30 min of SNP treatment, and GAPDH levels served as the loading control in western blotting. (C) Nitrosylated Hsp90 (SNO-Hsp90) was measured by western blotting after streptavidin agarose bead collection. The column scatter dot plot represents the mean values ± SEMs. The *p*-value was obtained by t test.


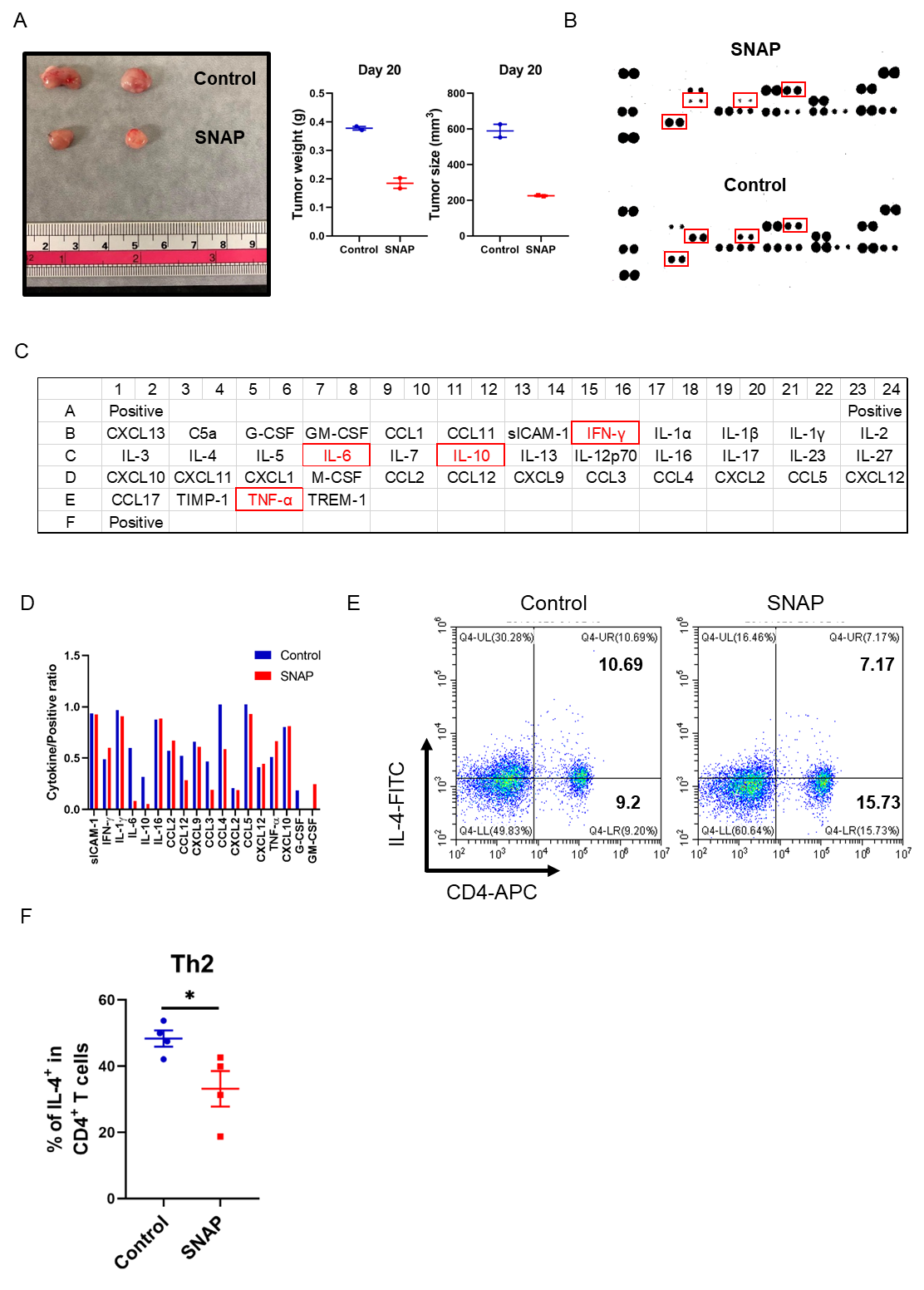


**Supplementary Figure 2: Low-dose SNAP regulates cytokine expression levels and the Th2 cell population in the spleen.**

(A) The tumor weight and tumor size on the day mice were sacrificed. Tumor tissues were isolated from LL2 tumor-bearing mice. The mice were sacrificed on the 20^th^ day after LL2 tumor implantation. (B) A cytokine array was used to determine the cytokine levels in the culture medium of splenocytes, which were co-cultured with LL2 cell lysates at 37 ℃ for 24 hr. The splenocytes were collected from 0.004 mg/kg SNAP treated mice (*upper*) and PBS-containing 0.004 % DMSO treated mice (*lower*). (C) The coordinates of every cytokine in the array. (D) The expression levels of each cytokine in the control and low-dose SNAP treatment groups. (E) The quadrants represent the percentage of CD4^+^ and/or IL-4-positive cells. (F) The percentage of IL-4-positive cells in the CD4^+^ population (type 2 T helper cells; Th2). LL2 tumor-bearing mice were administered SNAP (0.004 mg/kg) at 11 to 13 and 16 to 18 days, and splenocytes were isolated and analyzed. The column scatter dot plot represents the mean values ± SEMs. **p* < 0.05, versus the control group. ns, no significant difference. *P*-values of the Th2 percentages were obtained by t test.


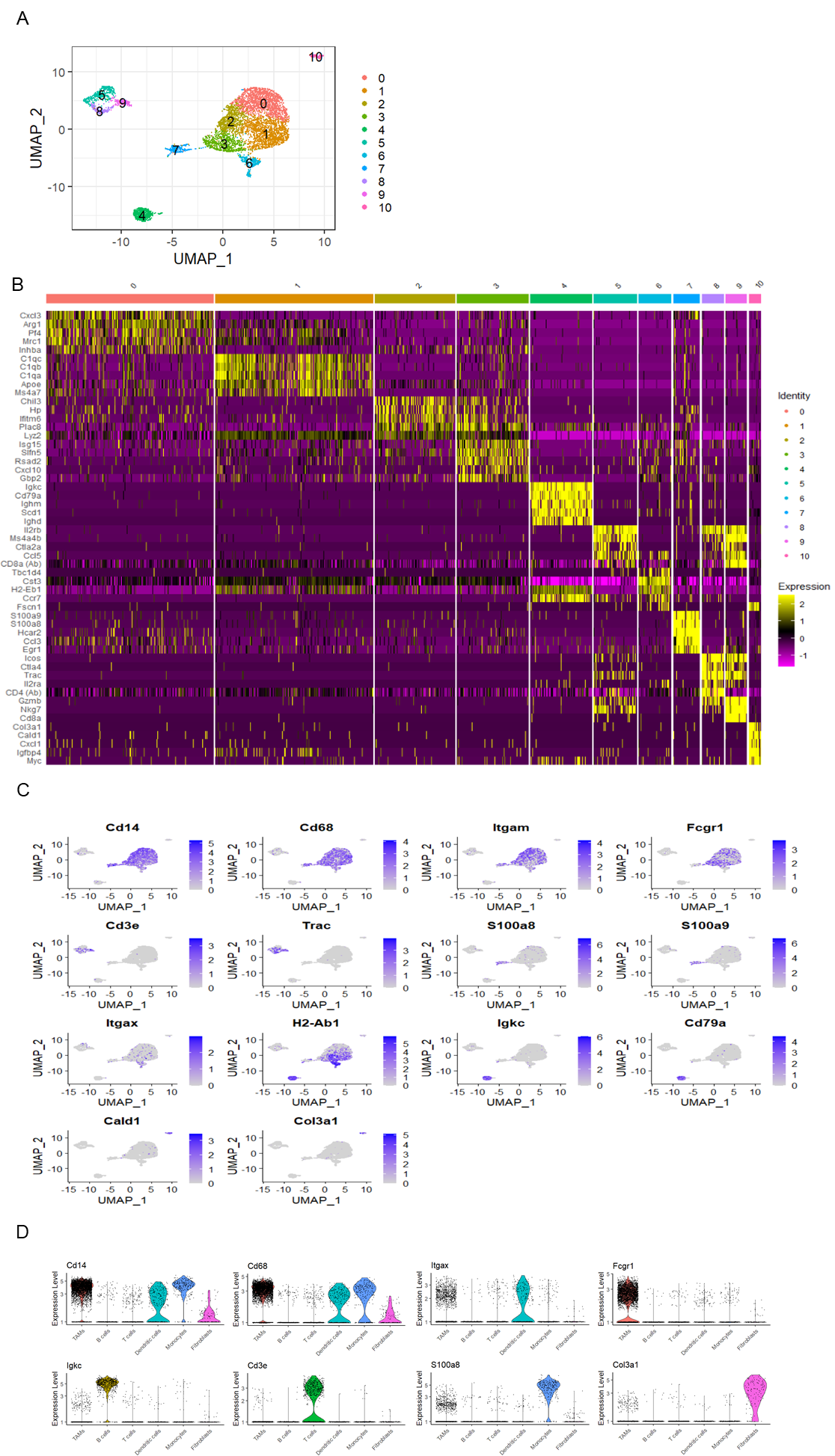


**Supplementary Figure 3: Reduction of cell clusters from 11 to 6 by the levels of marker genes expressed in CD45^+^ cells.**

(A) Clustering of CD45^+^ and subset visualization. UMAP dimensionality reduction of total CD45^+^ cells (6563 cells) was executed based on visualization of relevant cell clusters. All clusters were determined using the “FindCluster” function in the Seurat package. (B) Heatmap displaying the top 5 differentially expressed genes (DEGs) and mRNA levels in 11 clusters, and the top five DEGs list is presented in Supplementary Data 1. (C) Feature plot depicting single-cell gene expression of marker genes. *Cd14*, *Cd68*, and *Itgam* are marker genes for tumor-associated macrophages (TAMs). Cd3e and Trac were used for T-cell identification. *S100a8*, *S100a9* and *Fcgr1* were used to identify monocytes (monocytes did not express *Fcgr1*). Dendritic cells were identified by *Itgax* (CD11c) and *H2-Ab1* (MHC II molecule). B cells expressed the *Igkc* and *Cd79a* genes. *Cald1* and *Col3a1* were expressed on fibroblasts. (D) Violin plot showing the expression levels of each marker gene in each cluster.


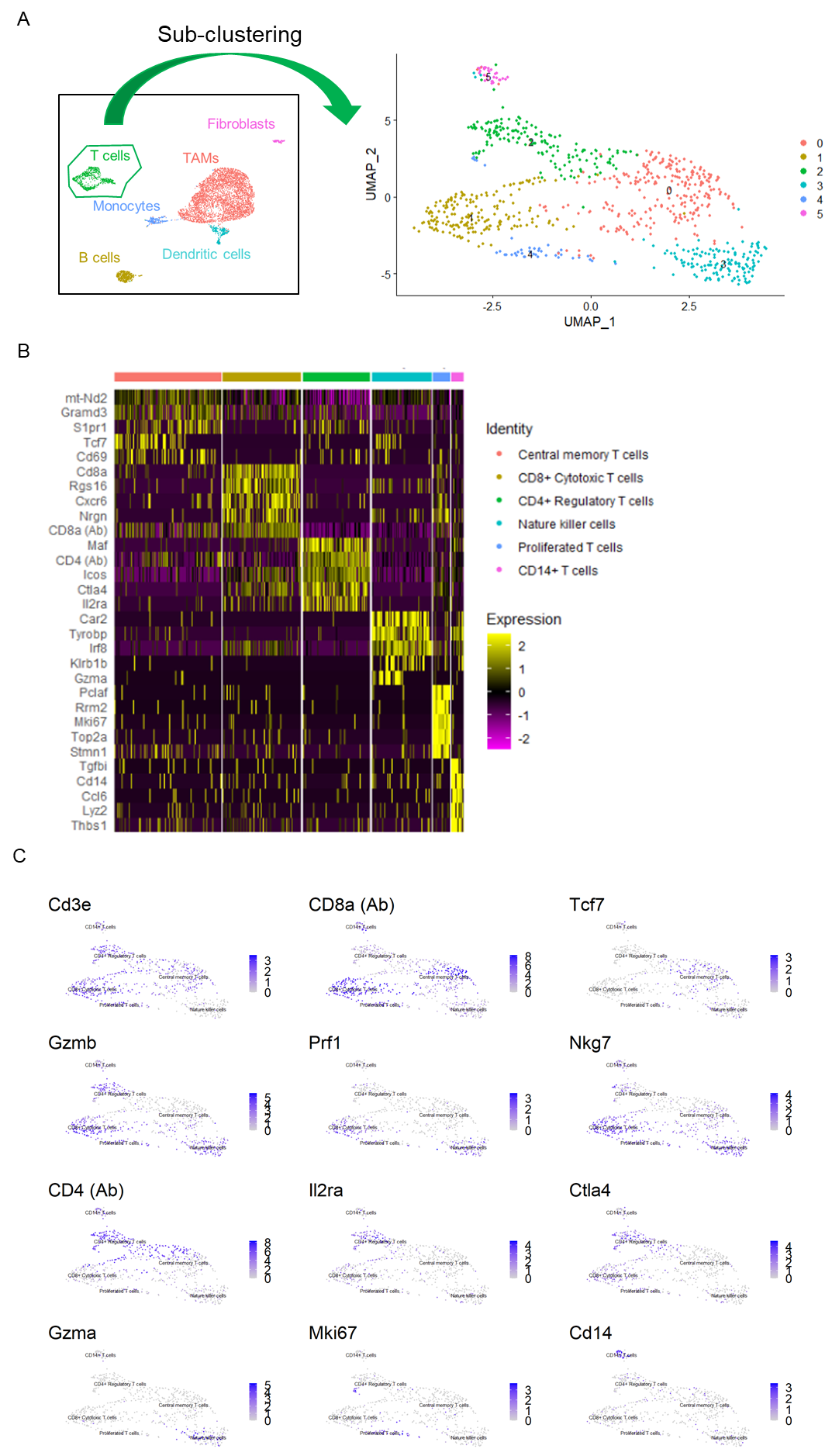


**Supplementary Figure 4: T lymphocytes are distinguished into 6 clusters and identified by marker genes.**

(A) Subclustering of T lymphocytes and visualization of the subsets. UMAP dimensionality reduction of 813 T lymphocytes (control: 463; drug: 350) was executed based on visualization of relevant cell clusters. All clusters were determined using the “FindCluster” function in the Seurat package. (B) Heatmap displaying the top 5 DEGs and mRNA levels in six subsets of T lymphocytes, and the gene list is shown in Supplementary Data 2. (C) Feature plot showing the single-cell gene expression of marker genes. Cd3e represents T cells. CD8a (Ab) and *Cd8a* are marker genes of CD8^+^ T cells. *Gzma*, *Gzmb*, *Prf1*, and *Nkg7* were used to identify the cytotoxic function of cells. *Tcf7* is an essential marker of central memory T cells. CD4 (Ab) was used to identify CD4^+^ T cells, and *Il2ra* (CD25) and Ctla4 determined the regulatory function of CD4^+^ T cells. *Mki67* is a proliferation marker.


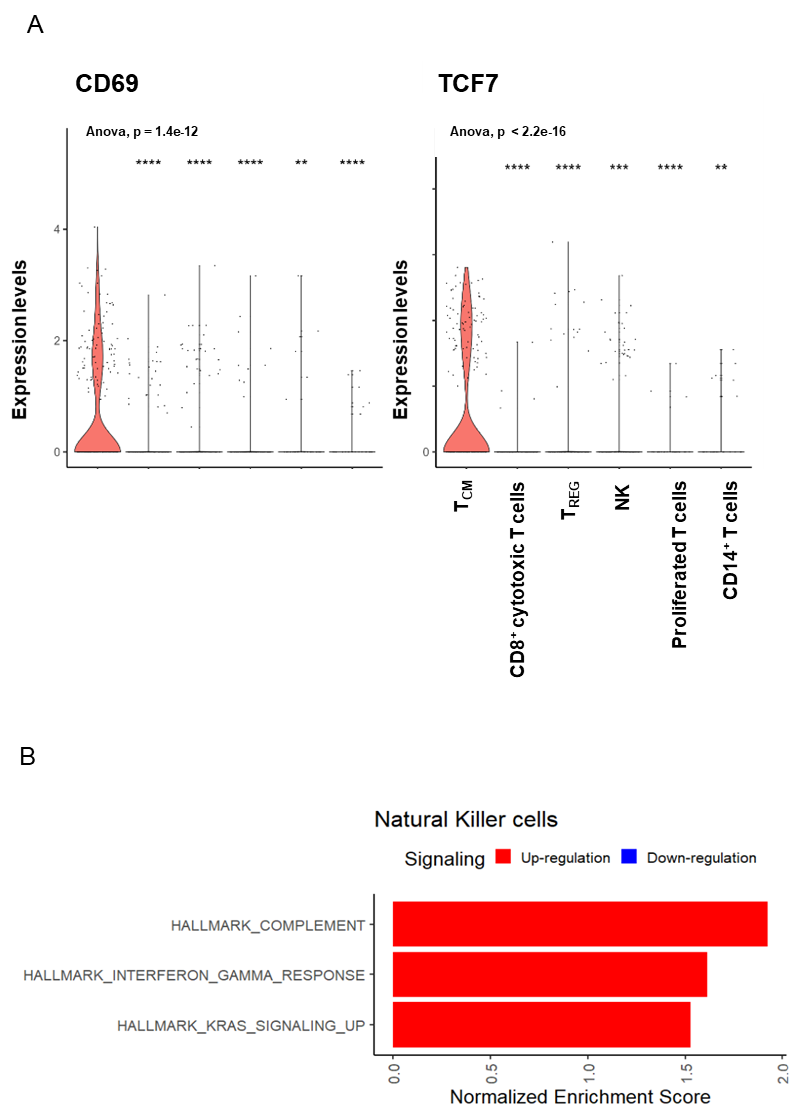


**Supplementary Figure 5: TCF7 and CD69 expression levels in T-cell subsets and GSEA analysis of NK cells.**

(A) Violin plot showing *Tcf7* and *Cd69* expression levels in T-cell subsets. (B) MSigDB Hallmark gene set displayed significant overlap with the DEGs of natural killer cells. The DEGs of NK cells were identified by the “FindMarker” function in the Seurat package and the DEGs list was shown in supplementary data 4. *p*-values of the violin plot were obtained by ANOVA. ***p* < 0.01, ****p* < 0.001, *****p* < 0.0001 versus the TAM1 group.


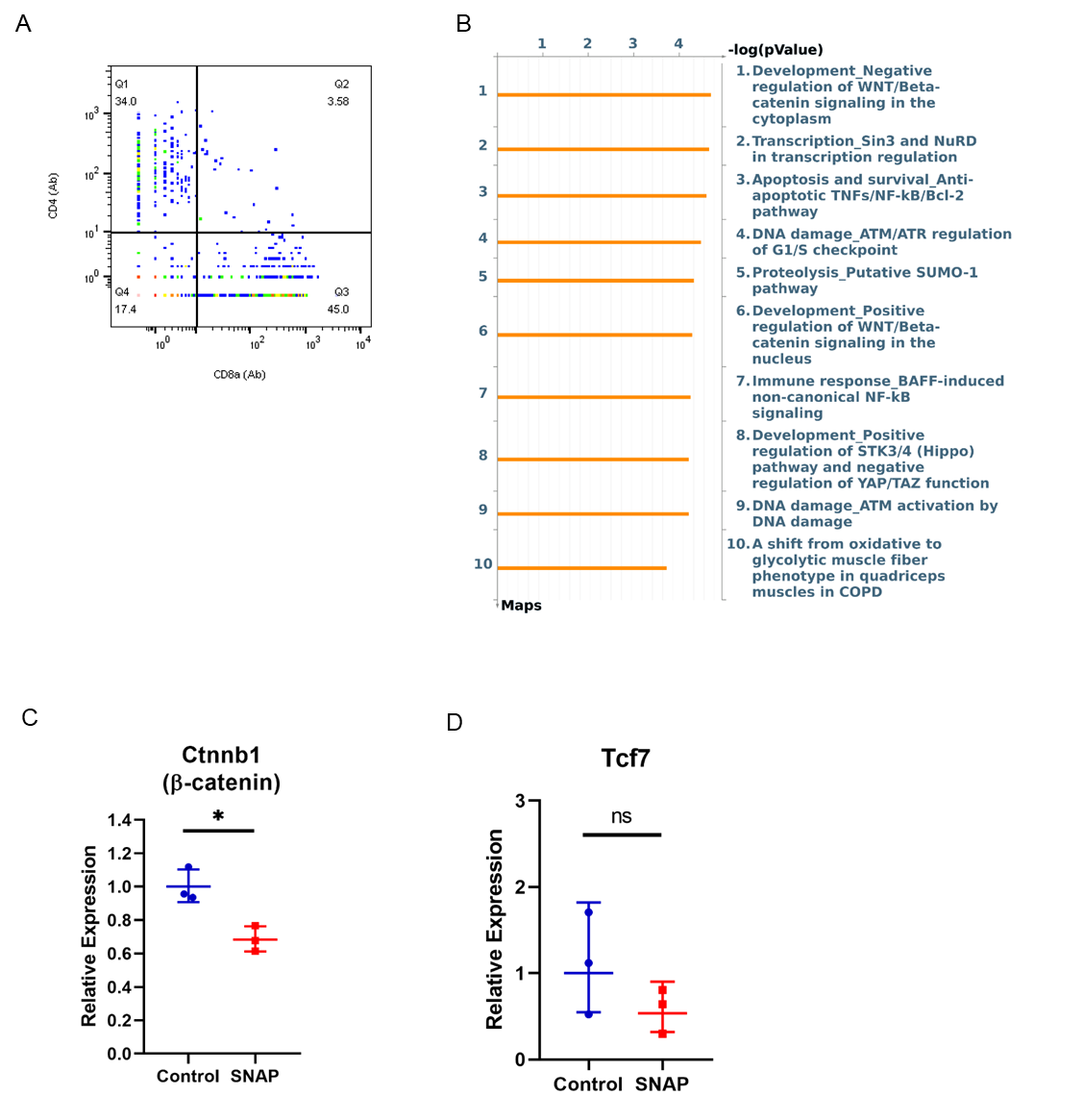


**Supplementary Figure 6: Low-dose SNAP regulates the Wnt/beta-catenin pathway in CD8^+^ T cells.**

(A) T lymphocytes were identified by CD4 (Ab) and CD8 (Ab) Abseq antibodies. Q3 are designated as CD8^+^ T cells. (B) Bar chart showing the significant pathways in CD8^+^ T cells after low-dose SNAP treatment. Significant genes were selected for pathway analysis, and the gene list is shown in Supplementary Data 5 (*p*-value < 0.05, fold change > 1.5, percentages of expressed cells > 10, and the *p*-value was obtained by t test). (C) Ctnnb1 (beta-catenin) and (D) Tcf7 mRNA levels in intratumoral CD8^+^ T cells from the control and 0.004 mg/kg SNAP treatment groups as determined by RT‒qPCR. The column scatter dot plot represents the mean values ± SEMs. *p*-values of the column scatter dot plot were obtained by t test. **p* < 0.05, ns, no significant difference.


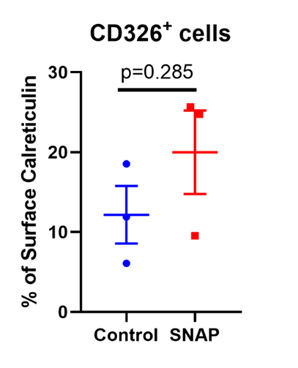


**Supplementary Figure 7: Low-dose SNAP treatment did not significantly induce the expression of surface calreticulin in LL2 tumor-bearing mice.**

Flow cytometry analysis of surface calreticulin in CD326-positive tumor cells isolated from LL2 tumor-bearing mice between the control and 0.004 mg/kg SNAP treatment groups. The mice were sacrificed at Day 20 after LL2 cell implantation. The column scatter dot plot represents the mean values ± SEMs. The *p*-values were obtained by t tests.


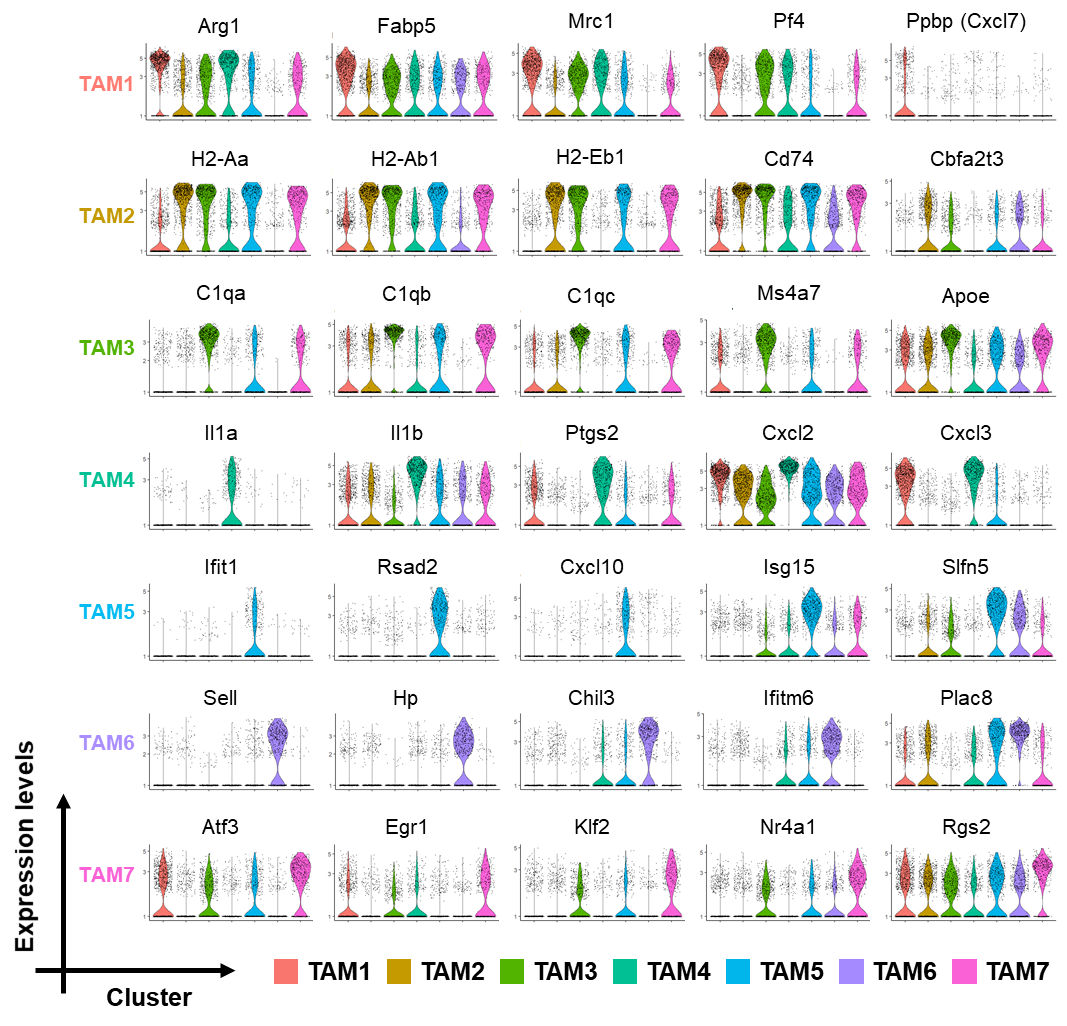


**Supplementary Figure 8: The mRNA levels of the top 5 differentially expressed genes in the TAM subsets.**

Violin plot demonstrating the expression levels of the top five DEGs in each subset of TAMs. The Y-axis represents log-normalized expression levels.


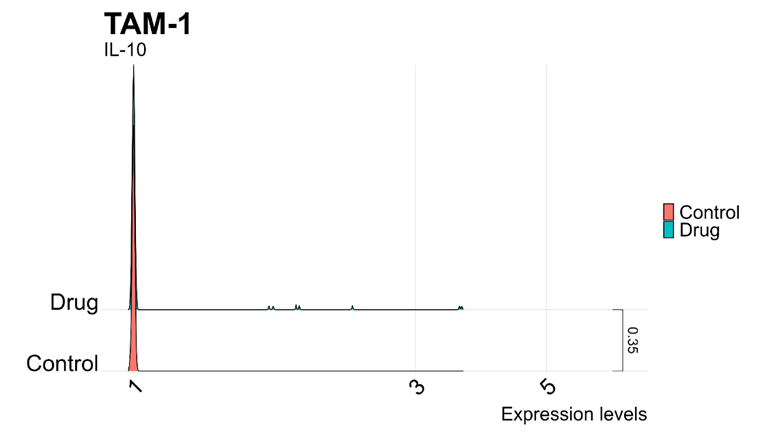


**Supplementary Figure 9: TAM1 cells do not express the immunosuppressive cytokine IL-10.**

Ridge plots determining IL-10 expression in the TAM1 subset. The X-axis represents log-normalized expression levels. *p*-values of the violin plot were obtained using the Wilcoxon test. ns, no significant difference.


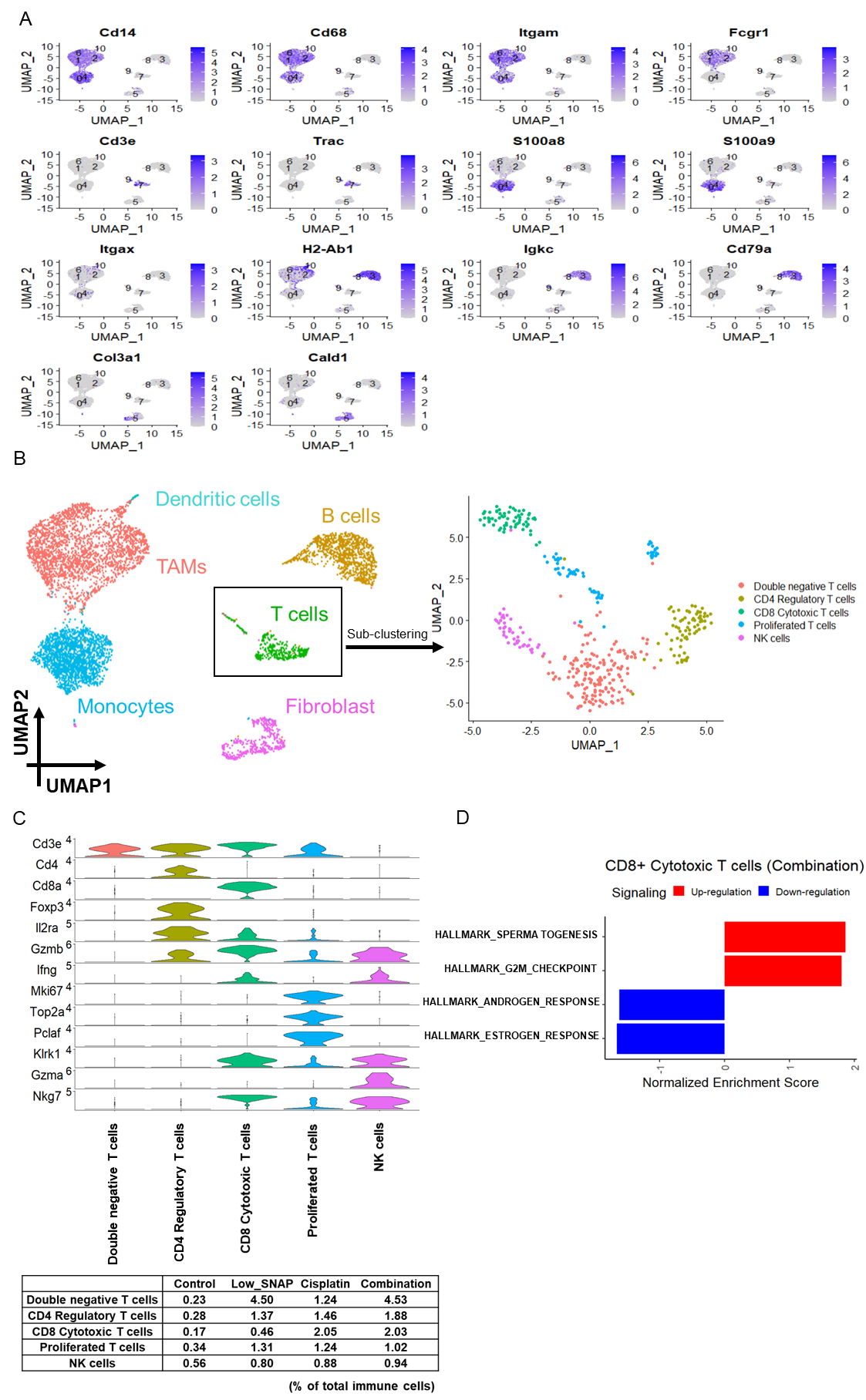


**Supplementary Figure 10: Single-cell RNA sequencing analysis of combination cisplatin and low-dose SNAP treatment.**

(A) Feature plot demonstrating the gene expression of marker genes. *Cd14*, *Cd68*, and *Itgam* are marker genes for tumor-associated macrophages (TAMs). *Cd3e* and *Trac* were used for T-cell identification. *S100a8*, *S100a9* and *Fcgr1* were used to identify monocytes (monocytes did not express *Fcgr1*). Dendritic cells were identified by *Itgax* (CD11c) and *H2-Ab1* (MHC II molecular). B cells expressed the genes *Igkc* and *Cd79a*. *Cald1* and *Col3a1* were expressed on fibroblasts. (B) *Left panel*: Clustering of intratumoral CD45^+^ cells and visualization of each type of cell. Uniform manifold approximation and projection (UMAP) of single-cell RNA sequencing data from 6690 cells (Control: 1909; Low-dose SNAP: 1943, Cisplatin: 1474, and Drug Combination: 1364) revealed six clusters determined by 14 specific markers (Supplementary Fig. 2). *Right panel*: Subclustering of T lymphocytes and visualization of the subsets. UMAP dimensionality reduction of 403 T lymphocytes was executed based on visualization of relevant cell clusters. (C) *Upper panel*: Violin plots revealed the expression levels of the marker genes for the subset of T cells. The Y-axis represents log-normalized expression levels. *Cd3e* serve as a T-cell marker; *Foxp3*, *Il2ra*, and *Cd4* serve as CD4 regulatory T-cell markers; *Gzmb*, *Ifng*, and *Cd8a* serve as CD8 cytotoxic T-cell markers; *Mki67*, *Top2a*, and *Pclaf* serve as proliferating T-cell markers; *Klrk*, *Nkg7*, and *Gzma* serve as NK cell markers. *Lower* *panel*: The percentages of each group of the subset of T cluster within total immune cells. (D) GSEA of CD8^+^ cytotoxic T cell DEGs between combination and cisplatin treatment. The red bar chart and blue bar chart show upregulated and downregulated pathways in the combination treatment group, respectively.


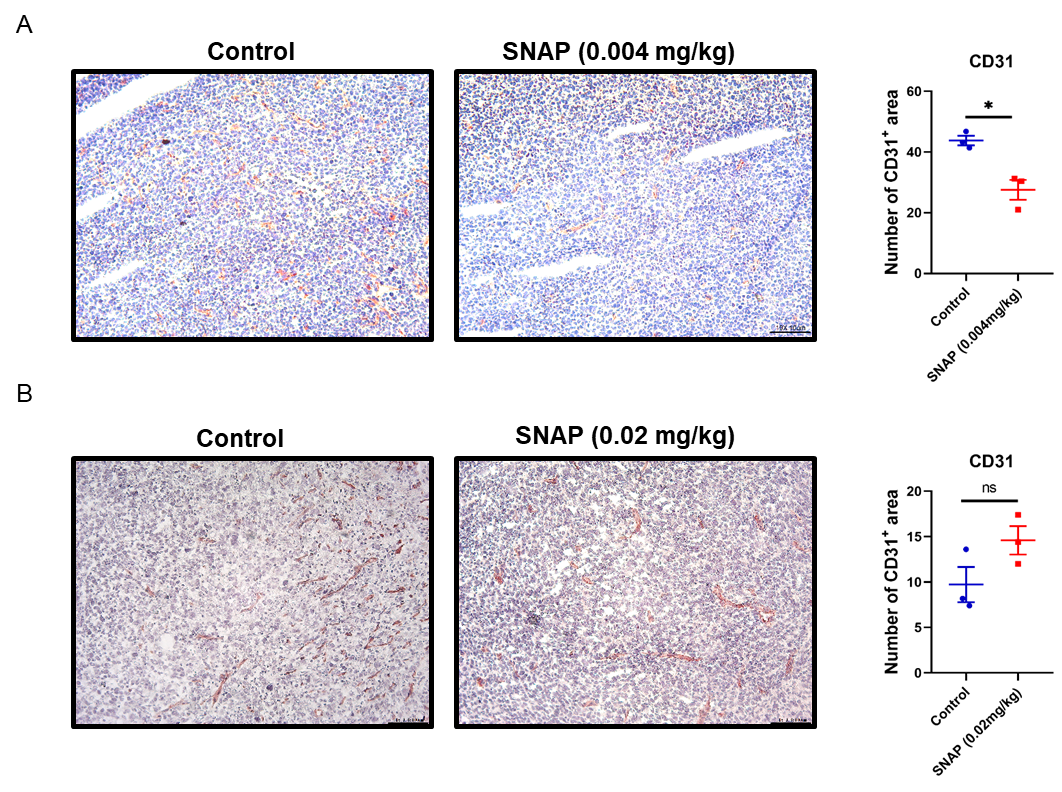


**Supplementary Figure 11: Treatment with 0.004 mg/kg but not 0.02 mg/kg SNAP decreased angiogenesis in the tumor microenvironment.**

(A) Frozen sections of 0.004 mg/kg or (B) 0.02 mg/kg SNAP-treated and control tumors from LL2 tumor-bearing mice stained with the angiogenesis marker CD31 (red). Five randomly chosen areas in each tumor sample were used to calculate the CD31^+^ area. The column scatter dot plot represents the mean values ± SEMs. **p* < 0.05, ns, no significant difference. *p*-values of the column scatter dot plot were obtained by t test.
